# Metabolic Profiling in Blastocoel Fluid and Blood Plasma of Diabetic Rabbits

**DOI:** 10.3390/ijms21030919

**Published:** 2020-01-30

**Authors:** Maria Schindler, Sophia Mareike Pendzialek, Katarzyna Grybel, Tom Seeling, Anne Navarrete Santos

**Affiliations:** Institute of Anatomy and Cell Biology, Martin Luther University Faculty of Medicine, Grosse Steinstrasse 52, D-06097 Halle (Saale), Germany; sophia_mareike.pendzialek@uni-leipzig.de (S.M.P.); katarzyna.grybel@wp.pl (K.G.); tom.seeling@medizin.uni-halle.de (T.S.); a.navarrete-santos@medizin.uni-halle.de (A.N.S.)

**Keywords:** Metabolomics, diabetic pregnancy, blastocoel fluid

## Abstract

Metabolic disorders of the mother adversely affect early embryo development, causing changes in maternal metabolism and consequent alterations in the embryo environment in the uterus. The goal of this study was to analyse the biochemical profiles of embryonic fluids and blood plasma of rabbits with and without insulin-dependent diabetes mellitus (DT1), to identify metabolic changes associated with maternal diabetes mellitus in early pregnancy. Insulin-dependent diabetes was induced by alloxan treatment in female rabbits 10 days before mating. On day 6 post-coitum, plasma and blastocoel fluid (BF) were analysed by ultrahigh performance liquid chromatography-tandem mass spectroscopy (UPLC-MS/MS) (Metabolon Inc. Durham, NC, USA). Metabolic datasets comprised a total of 284 and 597 compounds of known identity in BF and plasma, respectively. Diabetes mellitus had profound effects on maternal and embryonic metabolic profiles, with almost half of the metabolites changed. As predicted, we observed an increase in glucose and a decrease in 1,5-anhydroglucitol in diabetic plasma samples. In plasma, fructose, mannose, and sorbitol were elevated in the diabetic group, which may be a way of dealing with excess glucose. In BF, metabolites of the pentose metabolism were especially increased, indicating the need for ribose-based compounds relevant to DNA and RNA metabolism at this very early stage of embryo development. Other changes were more consistent between BF and plasma. Both displayed elevated acylcarnitines, body3-hydroxybutyrate, and multiple compounds within the branched chain amino acid metabolism pathway, suggesting that lipid beta-oxidation is occurring at elevated levels in the diabetic group. This study demonstrates that maternal and embryonic metabolism are closely related. Maternal diabetes mellitus profoundly alters the metabolic profile of the preimplantation embryo with changes in all subclasses of metabolites.

## 1. Introduction

During the preimplantation period, the development of the embryo is a well-regulated sequence of molecular events, starting with fertilisation of the oocyte and ending with implantation of the blastocyst in the uterine endometrium. The supply of certain key nutrients is essential for proper embryo development. Even though the developing embryos have the capacity and plasticity to deal with nutritional imbalances posed by an altered maternal metabolism, there is often a trade-off to the overall fitness of embryos later in life.

During preimplantation development, the preferred energy source for embryonic cellular metabolism changes [1,2]. In the early stages of embryonic development, aerobic glycolysis, also known as the Krebs cycle, predominates, using pyruvate and lactate as the main sources of energy. As development progresses, glucose uptake steadily increases, and glycolysis becomes increasingly more utilised for energy production [3]. At the blastocyst stage, glucose metabolism predominates as the main energy source. This increase in glucose consumption in late-stage preimplantation embryos has been observed across several species, including mice, rats, humans, cows, sheep, rabbits and pigs [4].

To evaluate early embryo development, the majority of research has quantified morphological status and miRNA and/or mRNA expression, as well as protein levels. However, analyses of the transcriptome and proteome provide information only on the effect on target gene expression, and little information regarding the overall effect on the dynamic metabolic status of the developing organism. Beside the proteome and transcriptome, the measurement of metabolites has begun to attract attention [5]. Metabolomics offers a new approach for global metabolite analysis. It is defined as the non-targeted identification and quantification of low molecular weight end-products of metabolism, so-called metabolites [6], and gives valuable information about the metabolism within cells [7]. In response to a disease state, the cells of an organism modify the concentration of numerous metabolites to maintain homeostasis [8]. Metabolites inside the cells are in dynamic balance with those in the surrounding milieu which perfuse the cells. Changes in metabolite profile within cells should be reflected in the composition of the surrounding milieu [9].

The analytical approaches for metabolomics are generally subdivided into either untargeted or targeted [10,11]. Both approaches have been used as a diagnostic tool for diseases in reproductive medicine [9]. On the one hand, it has been used for the prediction and diagnosis of gestational diabetes mellitus (GDM) to provide novel insights into the onset, severity and progression of GDM [12]; the main results indicate that carbohydrate, fatty acid and amino acid metabolism are impaired in infants and mothers in GDM-affected pregnancies [13,14,15]. On the other hand, metabolomics has been applied to the identification of non-invasive biomarkers for diagnostic and prognostic purposes in in vitro fertilisation (IVF) [9,16]. However, so far, the metabolic analysis of follicular fluid, embryo culture medium and/or blood plasma has revealed no single biomarker for IVF diagnostics, presumably because the metabolic picture is far more complex than can be explained by a single metabolite imbalance.

Therefore, a more comprehensive understanding of the reproductive tract environment and metabolism of the developing embryo can lay a foundation for comparative studies of abnormal development due to environmental insults. Insulin-dependent diabetes mellitus of the mother leads to an imbalance of maternal metabolism during preimplantation development, affecting the female reproductive system and the developing embryo [17,18]. Changes in metabolite profile within embryonic cells should be reflected in the composition of the blastocoel fluid (BF). The goal of this study was to interrogate biochemical profiles manifested in BF and plasma collected from rabbits with and without the chemical inducement of diabetes prior to pregnancy. To the best of our knowledge, this is the first study which linked metabolomics findings of embryonic biofluids to blood plasma of the mother in the context of maternal diabetes mellitus.

## 2. Results

**Figure 1:**
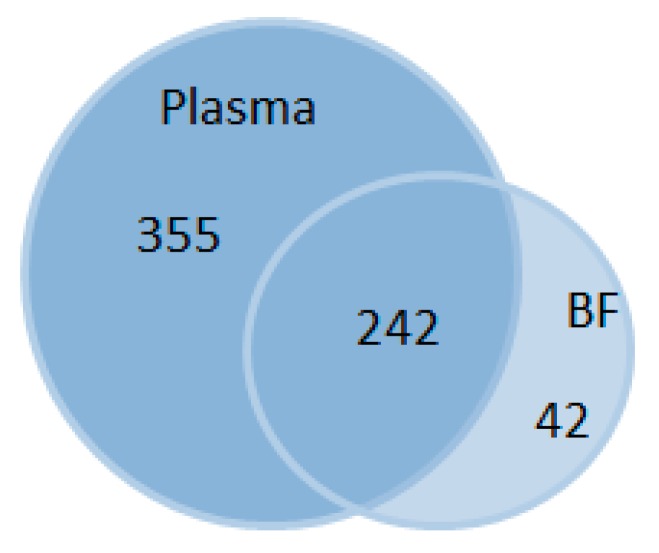
Venn diagram for total numbers of biochemicals in plasma and embryonic blastocoel cavity fluid (BF) in rabbits. 597 biochemicals were detected in total.

The present dataset comprises a total of 284 compounds of known identity (named biochemicals) in blastocyst cavity fluid and 597 in plasma (Figure 1, all detected biochemicals are listed in Supplementary Files).

The metabolic profiling of maternal plasma and BF demonstrated that maternal diabetes mellitus has a profound effect on both maternal and embryonic metabolism. A summary of the number of biochemicals that achieved statistical significance (*p* ≤ 0.05) is shown in Table 1.

Comparing diabetic vs. non-diabetic rabbits, we observed 339 biochemicals (57% of all biochemicals) that were changed in plasma due to experimentally-induced diabetes and 135 differences (48% of all biochemical) in embryonic samples. Most of the biochemicals were increased, plasma: ↑232 vs. ↓107 and BF: ↑117 vs. ↓18. Only 66 biochemicals were equally changed in plasma and BF, of which 58 biochemicals were increased and 8 biochemicals decreased. An overview of selected biochemicals is provided in the following results section, structured in (i) carbohydrates and energy metabolites, (ii) lipids, (iii) amino acids, (iv) purine and pyrimidine and (v) vitamins and cofactors. A comprehensive overview of all detected biochemicals is provided in supplemental data.

### 2.1. Carbohydrates and Energy Metabolites in Maternal Plasma and Blastocysts Cavity Fluid Samples from Diabetic Rabbits

Carbohydrates were measured in maternal plasma and BF samples from non-diabetic and diabetic rabbits (Table 2, Supplementary Materials Table S1). As expected from a diabetes model, glucose was elevated and 1,5-anhydroglucitol (1,5-AG) was decreased in the plasma of diabetic rabbits. Interestingly, there was no significant elevation of glucose in the BF.

**Figure 2:**
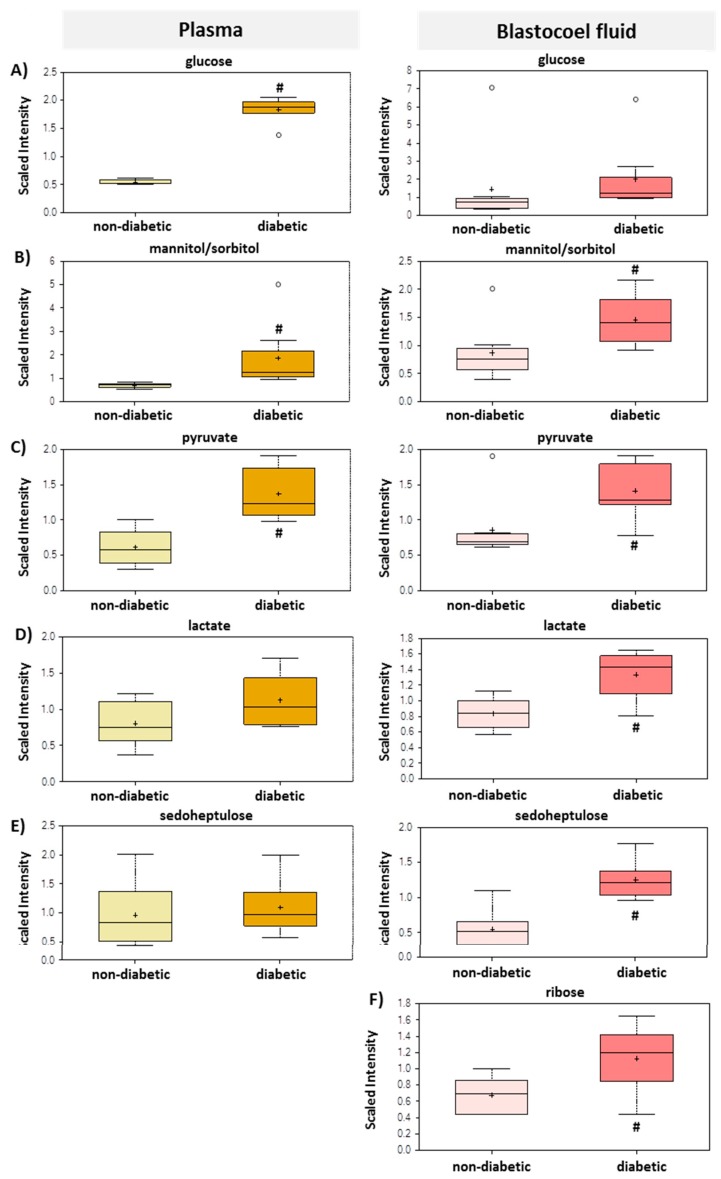
Selected biochemicals of carbohydrate metabolism detected in plasma and blastocoel fluid of non-diabetic and diabetic rabbits at day 6 post-coitum. Boxes in light yellow, red, plasma, and BF from non-diabetic rabbits, in dark yellow and red from diabetic rabbits. Box and whisker plots for biochemical components: (**A**) glucose, (**B**) mannitol/sorbitol, (**C**) pyruvate, (**D**) lactate, (**E**) seduloheptulose, (**F**) ribose. The upper whiskers represent the maximum, and the lower whiskers the minimum values. The plus-signs indicate the mean values, while the median values are represented by a black line within the boxes. Boxes with **#** differ significantly (*p* ≤ 0.05). y axis: scale intensity is calculated as followed: Each biochemical in OrigScale (raw data) is rescaled to set the median equal to 1.

Intermediates within the glycolysis pathway were also affected (Table 2, Figure 2). In plasma, pyruvate was significantly elevated and lactate was trending upwards (0.05 < *p* < 0.10), while both molecules were significantly elevated in BF. All metabolites belonging to pentose metabolism, like sedoheptulose, ribose and xylose, were increased in BF samples from diabetic rabbits, while only arabinose, arabitol/xylitol and arabonate/xylonate were increased in plasma.

Fructose and mannose were only present in maternal plasma and elevated in diabetic rabbits. Under hyperglycaemic conditions, glucose can be converted to sorbitol or form advanced glycation end products (AGEs). In both maternal plasma and BF, sorbitol levels were elevated in diabetic rabbits. The AGE, N6-carboxymethyllysine, was 2.65-fold increased in plasma, whereas it was not detectable in BF (Table 2).

Pyruvate, the end product of glycolysis, can enter the tricarboxylic acid cycle for ATP production. In plasma, we observed an increase in the early tricarboxylic acid cycle (TCA) intermediates citrate, aconitate, and succinylcarnitine (C4-DC). In BF, aconitate, isocitrate, α-ketoglutarate and succinylcarnitine (C4-DC) and the later intermediate malate were also elevated. The levels of phosphate were not affected in plasma and BF samples from diabetic rabbits (Table 2).

### 2.2. Lipid Metabolites in Maternal Plasma and Blastocysts Cavity Fluid Samples from Diabetic Rabbits

Lipid metabolites can be further divided into subgroups like fatty acids, eicosanoids, phosphatidylcholines, phosphatidylethanolamines, plasmalogens, diacylglycerols, sphingolipids and ceramides. In general, levels of almost all lipid metabolites were increased in the plasma and BF samples from diabetic animals, except for sphingolipids, amides and plasmalogens (Table 3, Supplementary Materials Table S2).

Medium, long chain and polyunsaturated fatty acids were only detected in plasma samples and not in BF samples (Table 3). All of the detected long chain and polyunsaturated fatty acids were increased in diabetic animals. Also, carnitine-bound fatty acids were elevated in plasma samples in elevated levels in diabetic rabbits. In BF samples, only short and medium chain carnitine-bound fatty acids were detectable. Nevertheless, all of the carnitine-bound fatty acids were increased or trended towards an increase. Carnitine levels were not changed (Table 3), however, the precursor of carnitine, deoxycarnitine, was increased in diabetic plasma and BF samples. Carnitine is necessary for the transport of fatty acids into the mitochondrion. Higher levels of carnitine-bound fatty acids also indicate a higher rate of fatty acid beta-oxidation. Increased levels of the ketone body 3-hydroxybutyrate (BHBA), a side product of beta-oxidation, in plasma and BF support this assumption (Table 3).

Prostaglandins and leukotrienes belong to the eicosanoid family and are derived from a common metabolic precursor, arachidonic acid. Elevated levels of the prostaglandins E2, A2 and F2α were detected in BF from the blastocysts of diabetic rabbits. The leukotriene 12-HETE was detectable in maternal plasma; it was not affected due to diabetes mellitus (Table 3).

Sphinganine is produced by the reaction of palmitoyl-CoA and serine and is the precursor for sphingolipid and ceramide synthesis. Sphinganine was only present in plasma and to a much lower level in plasma from diabetic rabbits (Table 3). Sphingolipid metabolites were also affected in the plasma of diabetic rabbits. Of the 38 metabolites in this pathway, 66% were significantly altered in diabetic rabbits, whereas with the exception of N-palmitoyl-sphinganine, all were decreased (Supplemental data). The remaining sphingolipids and ceramides were only detectable in plasma. 

Biochemicals of the phosphatidylethanolamine, phosphatidylinositol, phosphatidylcholine and lysophospholipid groups were only present in plasma, and most were elevated under diabetic conditions (Supplemental data). Also, plasmalogens and lysoplasmalogens were only detectable in maternal plasma (Supplemental data). However, almost all were reduced due to diabetes mellitus. The group of diacylglyerols are the biochemicals with the highest fold change in maternal plasma, with up to a 48.24-fold increase for linoleoyl-linelenoyl-glycerol (Table 3, Supplementary Materials Table S2). All diacylglycerols, which were detected, were significantly increased. In BF samples, diacylglycerols were not detectable.

Other classes of lipids are sterols and steroids, including cholesterol and cortisol (CEs). Cholesterol is synthesised from acetyl-CoA via the mevalonate pathway (Table 3). The precursor mevalonate was detected at elevated levels in BF samples and showed a trend towards being increased in the plasma of diabetic rabbits. Cholesterol was only detectable in plasma and was elevated under diabetic conditions (Table 3). Cortisol serves as a stress hormone and regulates intracellular metabolism. Levels of cortisol were only measurable in maternal plasma and were not changed by induced diabetes mellitus (Table 3).

### 2.3. Amino Acids and Their Derivatives in Maternal Plasma and Blastocyst Cavity Fluid Samples from Diabetic Rabbits

Amino acids and peptides were measured in maternal plasma and BF samples from non-diabetic and diabetic rabbits (Table 4, Supplementary Materials Table S3). Branched chain amino acids (BCAA; valine, leucine, isoleucine) were present in elevated levels in the diabetic group in both plasma and BF samples. The levels of BCAA derivatives, like 3-methyl-2-oxobutyrate, 3-methyl-2-oxovalerate, and 4-methyl-2-oxopentanoate, were also elevated in BF and plasma, indicating that BCAA metabolism is not impaired (Table 4).

Other important amino acids like glycine, serine, glutamine, histidine and lysine are reduced in the maternal plasma, but not affected, increased or tend to be reduced in BF samples. Phenylalanine and tyrosine, as well as their derivatives, are reduced in both BF and plasma. The levels of tryptophan were not significantly affected in maternal plasma or BF in diabetic rabbits. However, the tryptophan derivative serotonin was decreased by almost 80% in the plasma from diabetic rabbits. Methionine and arginine were not changed, while proline and alanine were increased in diabetic plasma samples (Table 4).

The concentration of creatine, a substrate for amino acid production, was increased in BF and plasma (Table 4). Creatine is formed by the methylation of guanidinoacetate, using S-adenosyl methionine as a methyl group donor, which was also elevated in both fluids. Creatine can further react with ATP or is metabolised to creatinine. Creatinine level was not affected in plasma, but increased in BF (Table 4), indicating that creatine is differently metabolised in maternal and embryonic tissues.

### 2.4. Purine- and Pyrimidine-related Biochemicals in Maternal Plasma and Blastocyst Cavity Fluid Samples from Diabetic Rabbits

Most of the biochemicals which belong to the purine and pyrimidine-related metabolites were not affected by insulin-dependent diabetes mellitus (Table 5, Supplementary Materials Table S4). However, biochemicals like allantoin, adenosine 5′-diphosphate (ADP) and dihydroorotate were significantly changed in plasma and BF from diabetic rabbits. Furthermore, urate and β-alanine were elevated, while adenine was reduced (Table 5).

### 2.5. Vitamins and Cofactors in Maternal Plasma and Blastocyst Cavity Fluid Samples from Diabetic Rabbits

Also, various vitamins and cofactors were affected in diabetic rabbits and embryos (Table 6, Supplementary Materials Table S5). Flavin adenine dinucleotide (FAD), which is a redox-active coenzyme associated with various proteins, was decreased, while retinol (vitamin A), α-tocopherol (vitamin E) and pantothenate (vitamin B5) were increased in plasma from diabetic rabbits. Pyridoxamine and pyridoxal, which are derivatives of vitamin B6, were reduced in plasma and BF samples. The vitamins α-tocopherol and its derivatives, retinol, thiamin and FAD were not measurable in BF. Biotin was only detected in BF, with a higher level in blastocysts from healthy rabbits (Table 6).

## 3. Discussion

The preimplantation blastocyst is supplied by the nutrients in uterine secretions. The trophoblast forms the nourishing and protective outer epithelium that surrounds the inner cell mass. The blastocyst cavity, the blastocoel, is filled with the fluid that is secreted by the trophoblast cells and contains cell metabolites, which to a certain extent provide substrates for the ICM. We demonstrated that insulin-dependent diabetes mellitus alters the maternal metabolism in the way that there is a surplus of metabolites in the blood. The increase in metabolites was also passed on to the embryo. In total, 66 metabolites were altered in the BF, similar to that seen in the maternal blood.

### 3.1. Maternal Diabetes Mellitus Increases Glycolysis and Pentose Metabolism in Preimplantation Blastocysts

As expected from a diabetes model system, glucose was elevated in the plasma of diabetic rabbits (Table 2). Interestingly, there was no significant elevation of glucose in the BF. This can be explained because the direct source of glucose in the BF is the surrounding tissue, the trophoblast, indicating that glucose is not transported through the trophoblast in significant amounts. The mammalian blastocyst expresses the facilitative glucose transporters (GLUTs) 1, 3, 4 and 8 in a specific orientation in the trophoblast cell membrane [19,20]. In mouse embryos, maternal hyperglycaemia induces a decrease in glucose uptake by the down-regulation of the GLUTs [21]. The regulation of GLUT1 has been observed in other cell types like skeletal muscle or retinal endothelial cells as well [22,23]. In previous studies of the diabetic rabbit model, glucose levels were quantified with a 4-fold and 3.5-fold increase in plasma and uterine secretions, respectively [18]. A down-regulation of GLUT1 in rabbit blastocysts could explain why the increased glucose level in plasma and uterine secretion does not correspond to BF.

**Figure 3:**
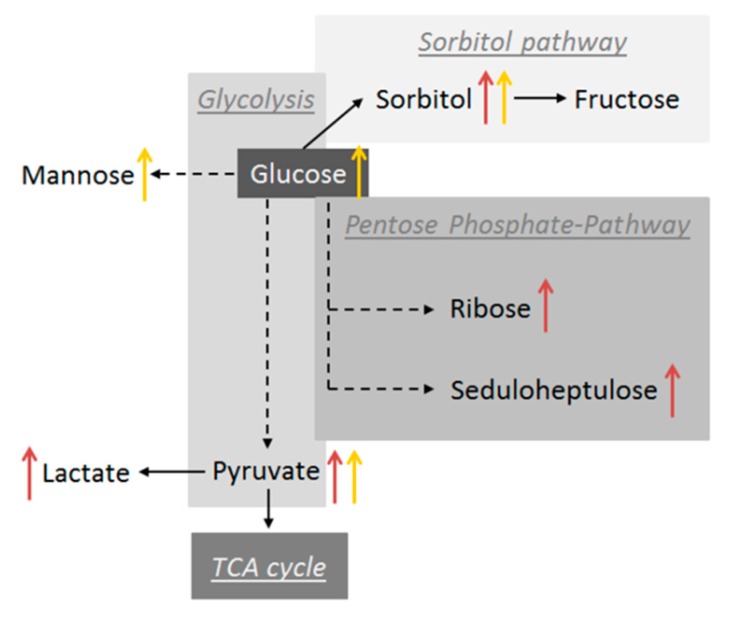
Schematic diagram of detected biochemicals in glycolysis and pentose phosphate pathway. Changes of metabolite concentration are indicated by arrows (↑), in yellow for diabetic plasma samples and in red for blastocoel fluid from 6 day old blastocysts of diabetic rabbit.

The fact that trophoblast cells explain a forced glucose metabolism becomes apparent by the increase in pyruvate and lactate, which were released into the BF in much higher amounts (Figure 2 and Figure 3). In plasma, pyruvate is significantly elevated and lactate is trending upwards (0.05 < *p* < 0.10), while both molecules are significantly elevated in BF (Figure 2). However, none of the downstream glycolytic intermediates were detected in either matrix, which suggests a very high level of glycolytic activity that is effectively keeping these intermediates at undetectable levels. The increase in lactate could possibly be due to increased oxidative glycolysis (Warburg metabolism), but is more likely a reflection of pyruvate accumulation due to the high levels of glycolysis. In human embryos, pyruvate can be converted to lactate and promotes early embryo development [24,25]. During preimplantation development, glucose, pyruvate and lactate serve as energy substrates [4,25]. Lactate is not only an energy source during preimplantation development, it is also a strong cytosolic reductant, via the activity of lactate dehydrogenase [26]. It alleviates the harmful effects of increased oxidative stress which has been described in diabetic pregnancies [27,28].

Besides glucose, 1,5-anhydroglucitol (1,5-AG) was also affected in diabetic rabbits. 1,5-AG is a validated marker of short-term glycaemic control. Poor glycaemic control is associated with lower serum 1,5-AG levels [29], as measured in diabetic rabbits. We demonstrate for the first time that 1,5-AG level was decreased in the embryos of diabetic mothers. Since de novo synthesis is relatively rare [29], it is tempting to speculate that 1,5-AG is taken up by blastocysts under physiological conditions. Based on cell culture experiments, it is known that 1,5-AG is hard to metabolise [30], indicating that is has no effect on intracellular metabolism. Whether altered 1,5-AG levels influence embryonic development needs to be clarified in further studies. 

In the current study, we could show that diabetes mellitus alters glucose metabolism in multiple ways (Figure 3). Under normal conditions, glucose is metabolised via the polyol pathway. In the presence of hyperglycaemia, high glucose levels saturate the hexokinase pathway and glucose is then metabolised by the polyol pathway [31]. We showed that embryonic sorbitol, mannitol and fructose concentrations were increased, indicating an elevated polyol pathway. This pathway is implicated in diabetic complications because of the consumption of NADPH and the harmful effects of sorbitol. Sorbitol cannot cross cell membranes and produce osmotic stress. Furthermore, sorbitol and fructose may also glycate nitrogens on proteins, such as collagen, producing advanced glycation end products (AGEs), like carboxymethyllysine (CML) and pentosidine [32]. We have recently shown that the total amount of AGEs was increased and that the level of CML was especially elevated in blastocysts from diabetic rabbits [33]. The metabolome analysis confirmed that N6-CML increased in plasma from diabetic rabbits.

The pentose phosphate pathway (PPP) is a major glucose catabolic pathway that links glucose metabolism to biosynthesis of the nucleotide precursor ribose and NADPH production. NADPH is an essential reductant in anabolic processes for the detoxification of intracellular reactive oxygen species [34]. Furthermore, NADPH is necessary for the reduction biosynthesis of fatty acids and steroid hormones and, as mentioned before, the sorbitol pathway [35]. In BF samples, all biochemicals belonging pentose metabolism were increased, presumably due to the need to produce more NADPH for antioxidant defence, in the same way as cancer cells [36]. However, in the bovine model, PPP activity was significantly increased in poor quality embryos [37]. The impact of activated PPP in the context of a maternal diabetes mellitus cannot be answered in the present study and needs to be elucidated in the future.

### 3.2. Diabetic Dysregulation of Maternal and Embryonic Lipid Metabolism

When glucose is in abundance, it can be metabolised and stored intracellularly, e.g., as lipids. In blastocysts from diabetic rabbits, the amount of lipid droplets increases [38], accompanied by elevated lipid plasma levels, supporting the assumption that lipid metabolism is favoured in the diabetic environment. According to the metabolic profiles, the amount of almost all fatty acids increased in the plasma of diabetic rabbits. This overload may lead to problems in embryo development, since excessive free fatty acids induce apoptosis by lipotoxicity [39]. Furthermore, carnitine-bound fatty acids were elevated in plasma. Acylcarnitines are critical for mitochondrial function, specifically for the beta-oxidation of fatty acids, and are used as early diagnostic markers of endocrine disorders [40]. They are synthesised by carnitine palmitoyltransferase 1 (CPT1), which is responsible for the transportation of fatty acids into the mitochondrial matrix [41]. Incomplete fatty acid oxidation results in elevated acylcarnitine concentrations [42] as observed in plasma from diabetic rabbits. In BF, short chain carnitine-bound fatty acids, like acetylcarnitine, 3-hydroxybutyrylcanitine and hexanoylcarnitine, were also increased, indicating impaired embryonic beta-oxidation as well. We have recently shown that CPT1 is increased in blastocysts from diabetic rabbits [43], indicating increased fatty acid oxidation in blastocysts from diabetic rabbits. This result is in line with the observed elevated BHBA levels in maternal plasma and BF due to diabetes mellitus. BHBA is a ketone body which is synthesised via the oxidation of acetyl-CoA and increased in diabetic milieu [44,45]. Elevated levels of ketone bodies can generate oxygen radicals and cause lipid peroxidation [44] and may have harmful effects for preimplantation development. In mouse studies, the culture of preimplantation embryos with ketone bodies led to marked embryo retardation [46]. The level of carnitine, the beta-oxidation cofactor, was not affected in maternal plasma and BF due to diabetes mellitus. The precursor, deoxycarnitine, however, was increased in both biofluids, indicating an altered carnitine biosynthesis under diabetic conditions. Adding carnitine in non-toxic concentrations to embryo culture medium decreases DNA damage and improves the in vitro blastocyst development rate, as well as the implantation rate of mammalian embryos [47,48,49,50]. Thus, optimal levels of carnitine are fundamental for preimplantation development. 

Fatty acids can be shunted to more complex lipids like phospholipids, sphingolipids and eicosanoids. Multiple phospholipids, including 1-stearoyl-2-arachidonoyl-GPC, were elevated in the plasma of diabetic rabbits. This key phospholipid is an important activator of the p38 MAPK pathway under conditions of cell stress and lipotoxicity [51]. In blastocysts from diabetic rabbits, the p38 MAPK pathway is activated [43], indicating that high 1-stearoyl-2-arachidonoyl-GPC levels activate p38 MAPK pathway in embryonic cells.

Sphingolipids and ceramides were the only groups of lipid-related biochemicals that were decreased in maternal plasma due to diabetes mellitus. Regulation of the sphingolipid and ceramide pathway affects angiogenesis and impairs endothelial function and the remodelling of maternal spiral arteries that occurs in preeclampsia [52,53]. Preeclampsia is associated with diabetes mellitus [54,55]. Altered levels of sphingolipids and ceramides may contribute to the implantation failure that has been observed in diabetic pregnancy [56].

### 3.3. Amino Acid Metabolism is Differently Regulated in the Maternal Tissues and Embryo

Changes in BCAAs (valine, leucine, isoleucine) have been associated with the development of diabetes [57,58] and diabetic pregnancy [59,60]. Diabetic increases in BCAA levels activate mTOR-signalling and forces the synthesis of embryonic proteins [59]. Metabolites of the BCAA pathways enter the TCA at different points [61]. We believe that the oxidation of amino acids, and especially BCAAs, becomes an alternative energy source in diabetic conditions.

As well as for energy production, amino acids are used for protein synthesis, nitrogen supply and pH and osmotic regulation [62,63,64]. In particular, taurine and glycine function in osmotic regulation, since the fluid in the oviduct and uterus possess a lower salt content than serum, but the osmolarity is the same [64]. In vitro studies have further suggested that amino acids can act as oxidants, buffer intracellular pH in embryonic cells, participate in the differentiation of the embryo and regulate implantation [62,65,66]. Changes in amino acids can therefore have profound effects on early embryo development.

The amino acids aspartate, glutamine, 2-aminoadipate, N-acetyltryptophan, taurine, hypotaurine, N-acetyltaurine, and 5-methylthioadenosine (MTA) were changed in the opposite way when comparing plasma and BF. These 8 biochemicals from the 597 measured were the only ones where an opposite regulation pattern in plasma and BF was observed. Glutamine is an energy source for preimplantation embryos via the TCA, particularly after the 8-cell stage [67] and improves blastocyst formation in several mammalian species [68,69,70]. Furthermore, it serves as a substrate for glutathione synthesis, which is necessary to maintain redox balance and for embryo differentiation [65,71]. In BF, glutamine levels were increased, while lower levels of glutamine were observed in plasma, indicating that the embryo relies on glutamine especially as an energy source and glutathione supply in diabetic pregnancies. Also, taurine and its derivatives hypotaurine and N-acetyltaurine were increased in BF and decreased in plasma. Taurine can act as an osmolyte by being released from embryos in the hypoosmotic state [72] and taurine, as well as hypotaurine, improves the development of mammalian embryos [73,74]. Both glutamine and taurine were found in high concentrations in the reproductive tract [75,76,77,78].

The most-abundant amino acid in the human oviductal and uterine fluid is glycine [75,77]. Like taurine, glycine is important to maintain intracellular osmolarity [79,80]. Since early preimplantation embryos are particularly sensitive to changes in osmolarity [79,80], an optimal osmolarity is mandatory. Therefore, changes in glycine and taurine levels, as observed in blastocysts from diabetic rabbits, can affect the preimplantation embryo development. Blastocysts from diabetic rabbits have a higher number of apoptotic cells [18]. Glycine supplementation decreases apoptosis and increases embryo cell numbers in mammalian embryos [81,82,83]. Glycine also participates in the regulation of pH, nucleotide synthesis and methylation [77,84,85]. Lower levels of glycine may therefore lead to epigenetic modifications in embryos, which have been described in diabetic pregnancies [86,87].

It has been suggested that cell metabolism forms the basis of some epigenetic mechanisms that change gene expression in early embryos. In mice transcriptomics and metabolomics analyses showed that a non-physiological metabolic milieu of maternal obesity sets the stage very early in pregnancy by altering the transcriptome of placenta progenitor cells in the trophoblast. This was associated with profound implications for placenta development and function [88]. In the rabbit diabetic model, brief exposure to maternal type-1 diabetes in the periconception window, until the blastocyst stage, is able to irreversibly malprogram the feto-placental phenotype [89]. In women with pre-gestational diabetes mellitus, placental growth is impaired [90]. It is tempting to speculate that these changes are in relation to an altered metabolome profile in early pregnancy. Furthermore, differences in several lysine metabolites were observed, especially acetylated lysine molecules [88]. They are commonly found in histone proteins, changing active gene expression or modulating protein function during metabolism [91,92]. Also, we observed altered levels of acetylated lysine molecules in plasma and BF samples from diabetic rabbits (Supplemental data), which may lead to long-term effects on gene expression patterns.

The preimplantation blastocyst relies entirely on the supply by nutrients and metabolites in the uterine fluid. This native environment is the primary source of the metabolites that a developing embryo needs, and is actively modified by the epithelium lining in the uterus in response to the embryo’s development, but also to changes in maternal metabolism [2]. In the current study, we did not analyse the uterine fluid due to methodical limitations. Therefore, we can only speculate whether observed changes in BF are due to selective adaptation mechanism in the uterine epithelium or trophoblast cells. Based on our recent work, we could show that in the uterus, especially the endometrium, but also in the trophoblast transporters for glucose, amino acids and lipids, as well as metabolic pathway molecules are affected in a diabetic pregnancy [18,38,59], indicating that on maternal and embryonic side adaptation mechanism are possible.

## 4. Materials and Methods

### 4.1. Alloxan Treatment

Experimental insulin-dependent diabetes (DT1) was induced in mature 18 to 20 week-old female non-pregnant rabbits (outbred ZIKA-hybrid New Zealand White) by alloxan treatment (Sigma-Aldrich, Taufkirchen, Germany), as described by [18]. Rabbits were maintained under diabetic conditions (blood glucose levels 15-25 mmol/l, daily insulin supplementation) as described by [43] for at least 10 days before mating. All animal experiments were performed in accordance with the principles of laboratory animal care and the experimental protocol was approved by the local ethics committee (Landesverwaltungsamt Dessau; reference number: 42502-2-812, 31 January 2011).

### 4.2. Collection of Plasma Samples and Blastocoel Fluids for Metabolomics Analysis

On day 6 post-coitum, blood plasma from 16 pregnant rabbits (8 diabetic and 8 non-diabetic) was analysed. The blood was taken using the standard venepuncture of the vena auricularis. Blood samples were collected in EDTA tubes and processed by centrifugation in order to separate plasma. Plasma samples were isolated, aliquoted and stored at −80 °C until further use. Afterwards, the animals were anaesthetised and killed for organ and embryo harvesting.

Embryo recovery was performed as described previously [93]. Briefly, rabbit blastocysts were taken from the uterus and washed three times in cold PBS. Individual blastocyst was placed on the dry and cold surface of a watch glass dishes. An excess PBS was removed by pipetting. Blastocysts’ surface was further dried by flint-free cellulose paper. Under the stereomicroscope, blastocysts were punctured with sterile micro-needles and fluids were taken up with a pipette from the watch glasses and stored at −80 °C. Overall, washing, drying, puncturing and the collection of fluids took less than 2 min. The procedure was highly standardized in all experimental and performed by well-experienced laboratory staff members. BF was snap-frozen in liquid nitrogen and stored at −80 °C until processing for metabolomics analysis. In total, 8 plasma samples with corresponding pooled blastocoel fluid samples (100 µl from 5 blastocysts, 20 µl each) were analysed (Supplementary Materials Figure S1). For all embryos, the diameter and stage were recorded. Blastocysts were characterised morphologically and grouped by gastrulation stage. For metabolomics analyses, gastrulation stages 1 and 2 were summarised by [94].

### 4.3. Metabolic Analysis

Global biochemical profiles were determined in BF and plasma collected from pregnant, non-diabetic and diabetic rabbits. For each group, EDTA plasma samples from 8 individual rabbits and pooled BF samples from blastocysts collected from the corresponding rabbits were analysed, whereby each BF sample was isolated from 5 blastocysts from one rabbit (schema of sample collection: Supplemental data). Blastocysts did not differ between sizes and stages (see Table 7).

Sample preparation, ultrahigh performance liquid chromatography-tandem, spectroscopy (UPLC-MS/MS) and bioinformatics analysis were performed by Metabolon Inc. (Durham, NC, USA). Briefly, the automated MicroLab Star^®^ from Hamilton Company (Franklin, MA, USA) was used for sample preparation. The resulting extract was divided into fractions for further analysis: two for analysis by two separate reverse phase (RP)/UPLC-MS/MS methods with positive ion mode electrospray ionisation (ESI), one for analysis by RP/UPLC-MS/MS with negative ion mode ESI and one for analysis by HILIC/UPLC-MS/MS with negative ion mode ESI. Metabolon’s hardware and software (Durham, NC, USA) was used for further raw data extraction, peak-identification, and data processing for QC and compound identification. Biochemicals which belong to the xenobiotic group were excluded from further analysis. We observed no difference regarding the occurrence of single biochemicals due to diabetes mellitus, in either plasma or BF samples. However, diabetes mellitus altered the proportion of single biochemicals in plasma and BF, respectively (Table 7).

### 4.4. Statistics

For metabolomics analyses, log transformation and Welch’s two-sample t-test was used to identify biochemicals that differed significantly between experimental groups. A summary of biochemicals that were detected is shown below. Those who achieved statistical significance (*p* ≤ 0.05) were indicated by dark green and red and those approaching significance (0.05< *p* <0.10) by light green and red. An estimate of the false discovery rate (*q*-value) was calculated to take into account the multiple comparisons that normally occur in metabolomics-based studies (*q* < 0.10).

### 4.5. Data Availability

The datasets generated and analysed during the current study are available from the corresponding author upon reasonable request.

## 5. Conclusions

In conclusion, metabolism is the key determinant of oocyte and preimplantation embryo development. Despite intense research over the last few decades, the understanding of how the embryo-maternal dialog affects embryonic metabolism is still not complete; in particular, in pathophysiological conditions, there are still too many unanswered questions. We have shown that diabetes mellitus profoundly affects maternal and embryonic metabolism with changes in all subclasses of metabolites. The metabolic profiles reflect the complex pictures in maternal and embryonic tissues, and how maternal diabetes mellitus affects the milieu of mammalian preimplantation embryos.

## Figures and Tables

**Table 1:** Number of biochemicals that were detected and quantified in plasma and blastocoel fluid (BF) of diabetic rabbits by Metabolon^®^ analysis. Alterations were calculated as fold change of diabetic vs. non-diabetic values. Welch’s two-sample t-test was used to identify biochemicals that differed significantly between experimental groups.

Number of Biochemicals					
	Total	Not Altered (*p* > 0.05)	Altered (*p* ≤ 0.05)	Diabetic Effect	
				Increased	Decreased
Blastocoel fluid	284	149	135	117	18
Plasma	597	258	339	232	107

**Table 2:** List of selected carbohydrates and energy metabolites that were detected in maternal plasma and blastocysts cavity fluid (BF) in non-diabetic and diabetic rabbits (n.d.= not detectable).

Sub Pathway	Biochemical Name	Plasma	BF
Glycolysis, Gluconeogenesis and Pyruvate Metabolism	1,5-anhydroglucitol (1,5-AG)	0.16	0.29
	glucose	3.35	1.38
	pyruvate	2.24	1.65
	lactate	1.40	1.60
Pentose Metabolism	ribose	n.d.	1.67
	xylose	1.24	1.47
	arabinose	1.79	1.82
	arabitol/xylitol	1.93	2.13
	arabonate/xylonate	1.72	1.60
	sedoheptulose	1.14	2.27
Fructose, Mannose and Galactose Metabolism	fructose	8.34	n.d.
	mannitol/sorbitol	2.66	1.68
	mannose	1.83	n.d.
Advanced Glycation End-product	N6-carboxymethyllysine	2.65	n.d.
TCA Cycle	citrate	1.20	1.69
	aconitate [cis or trans]	1.82	1.89
	isocitrate	n.d.	2.01
	alpha-ketoglutarate	0.92	1.25
	succinylcarnitine (C4-DC)	1.45	1.82
	succinate	1.20	1.51
	fumarate	1.38	2.07
	malate	1.28	1.91
Oxidative Phosphorylation	phosphate	0.90	0.98

Fold changes were calculated as diabetic vs. non-diabetic. Cells that achieved statistical significance (*p* ≤ 0.05) were indicated with dark green and red and those approaching significance (0.05 < *p* < 0.10) with light green and red.

**Table 3:** List of selected lipids that were detected in maternal plasma and blastocysts cavity fluid (BF) in non-diabetic and diabetic rabbits.

Sub Pathway	Biochemical Name	Plasma	BF
Fatty Acid Synthesis	malonylcarnitine	n.d.	1.86
Medium chain fatty acids	caprate (10:0)	0.73	n.d.
	laurate (12:0)	1.00	n.d.
Long Chain Fatty Acid	myristate (14:0)	2.34	n.d.
	palmitate (16:0)	3.16	n.d.
	stearate (18:0)	2.30	n.d.
	arachidate (20:0)	1.81	n.d.
Poly unsaturated fatty acids (n3 and n6)	eicosapentaenoate (EPA; 20:5n3)	4.49	n.d.
	docosapentaenoate (n3 DPA; 22:5n3)	8.57	n.d.
	docosahexaenoate (DHA; 22:6n3)	5.69	n.d.
	arachidonate (20:4n6)	3.48	n.d.
	docosapentaenoate (n6 DPA; 22:5n6)	2.82	n.d.
Fatty Acid, Amide	palmitic amide	0.30	n.d.
	oleamide	0.32	n.d.
Fatty Acid Metabolism (Acyl Carnitine)	acetylcarnitine (C2)	1.66	1.87
	3-hydroxybutyrylcarnitine (1)	4.12	5.05
	hexanoylcarnitine (C6)	1.43	2.25
	octanoylcarnitine (C8)	2.14	1.89
	laurylcarnitine (C12)	1.94	1.86
	myristoylcarnitine (C14)	1.80	n.d.
	palmitoylcarnitine (C16)	1.88	n.d.
	stearoylcarnitine (C18)	1.82	n.d.
	linoleoylcarnitine (C18:2)	3.26	n.d.
	arachidonoylcarnitine (C20:4)	2.37	n.d.
Carnitine Metabolism	deoxycarnitine	1.56	1.66
	carnitine	1.05	1.19
Ketone Bodies	3-hydroxybutyrate (BHBA)	4.44	2.97
Eicosanoid	prostaglandin E2	n.d.	1.84
	prostaglandin A2	n.d.	1.83
	prostaglandin F2alpha	n.d.	1.60
	12-HETE	0.85	n.d.
Phospholipid Metabolism	choline	1.04	1.10
	choline phosphate	0.41	0.33
	1-palmitoyl-2-docosahexaenoyl-GPE (16:0/22:6)	3.67	n.d.
	1-stearoyl-2-linoleoyl-GPE (18:0/18:2)	1.60	n.d.
	1-stearoyl-2-arachidonoyl-GPE (18:0/20:4)	1.72	n.d.
	1-stearoyl-2-docosahexaenoyl-GPE (18:0/22:6)	11.63	n.d.
Phosphatidylinositol (PI)	1-stearoyl-2-arachidonoyl-GPI (18:0/20:4)	1.95	n.d.
Phosphatidylcholine (PC)	1-palmitoyl-2-linoleoyl-GPC (16:0/18:2)	1.60	0.80
	1-palmitoyl-2-arachidonoyl-GPC (16:0/20:4n6)	1.65	n.d.
	1-stearoyl-2-arachidonoyl-GPC (18:0/20:4)	1.29	n.d.
	1-linoleoyl-2-arachidonoyl-GPC (18:2/20:4n6)	1.64	n.d.
	1-stearoyl-2-docosahexaenoyl-GPC (18:0/22:6)	1.90	n.d.
Lysophospholipid	1-linoleoyl-GPA (18:2)	1.69	n.d.
	1-linoleoyl-GPC (18:2)	1.57	n.d.
	1-linolenoyl-GPC (18:3)	2.05	n.d.
	1-arachidonoyl-GPC (20:4n6)	1.83	n.d.
	1-palmitoyl-GPE (16:0)	1.44	n.d.
	1-stearoyl-GPE (18:0)	1.79	n.d.
	1-linoleoyl-GPE (18:2)	2.14	n.d.
	1-arachidonoyl-GPE (20:4n6)	1.92	n.d.
	1-stearoyl-GPS (18:0)	0.31	n.d.
	1-palmitoyl-GPG (16:0)	1.51	n.d.
Plasmalogen	1-(1-enyl-palmitoyl)-2-oleoyl-GPE (P-16:0/18:1)	0.37	n.d.
	1-(1-enyl-palmitoyl)-2-linoleoyl-GPE (P-16:0/18:2)	0.56	n.d.
	1-(1-enyl-palmitoyl)-2-arachidonoyl-GPE (P-16:0/20:4)	0.39	n.d.
	1-(1-enyl-palmitoyl)-2-oleoyl-GPC (P-16:0/18:1)	0.63	n.d.
	1-(1-enyl-palmitoyl)-2-linoleoyl-GPC (P-16:0/18:2)	0.74	n.d.
Glycerolipid Metabolism	glycerol	1.44	n.d.
Diacylglycerol	linoleoyl-linolenoyl-glycerol (18:2/18:3)	48.24	n.d.
Sphingolipid Metabolism	sphinganine	0.38	n.d.
	sphinganine-1-phosphate	0.81	n.d.
Mevalonate Metabolism	mevalonate	1.26	1.45
Sterol	cholesterol	1.69	n.d.
Corticosteroids	cortisol	1.23	n.d.
Primary Bile Acid Metabolism	cholate	0.62	n.d.
	glycocholate	1.96	n.d.
Secondary Bile Acid Metabolism	deoxycholate	0.62	0.59

Fold changes were calculated as diabetic vs. normoinsulinaemic. Cells that achieved statistical significance (*p* ≤ 0.05) were indicated with dark green and red and those approaching significance (0.05 < *p* < 0.10) with light green and red.

**Table 4:** Relative amounts of selected amino acids and peptides that were detected in maternal plasma and blastocoel fluid (BF) of diabetic rabbits.

Sub Pathway	Biochemical Name	Plasma	BF
Glycine, Serine and Threonine Metabolism	glycine	0.69	0.90
	N-acetylglycine	1.47	1.15
	serine	0.87	1.02
	N-acetylserine	1.51	0.87
	threonine	1.16	1.17
	N-acetylthreonine	1.90	1.32
Alanine and Aspartate Metabolism	alanine	1.41	2.01
	N-acetylalanine	1.48	1.41
	aspartate	0.74	1.60
	N-acetylaspartate (NAA)	0.66	0.83
	asparagine	0.99	0.91
	N-acetylasparagine	1.15	1.19
Glutamate Metabolism	glutamate	0.94	2.20
	glutamine	0.68	1.97
	N-acetylglutamate	1.13	1.59
	N-acetylglutamine	1.11	1.87
Histidine Metabolism	histidine	0.72	0.90
	1-methylhistidine	0.70	1.27
	3-methylhistidine	1.03	1.44
	N-acetylhistidine	1.07	n.d.
	histamine	0.18	n.d.
Lysine Metabolism	lysine	0.81	1.02
	N6-acetyllysine	1.54	1.86
	N2,N6-diacetyllysine	0.50	n.d.
	N6-formyllysine	2.74	2.31
	2-aminoadipate	1.61	0.61
Phenylalanine Metabolism	phenylalanine	0.84	0.94
	N-acetylphenylalanine	0.36	1.28
	phenylpyruvate	0.97	0.36
Tyrosine Metabolism	tyrosine	0.59	0.73
	N-acetyltyrosine	0.44	1.08
	thyroxine	1.02	n.d.
Tryptophan Metabolism	tryptophan	0.91	1.21
	N-acetyltryptophan	0.33	1.54
	serotonin	0.20	n.d.
Leucine, Isoleucine and Valine Metabolism	leucine	1.66	2.20
	N-acetylleucine	1.37	1.97
	isoleucine	1.57	2.04
	N-acetylisoleucine	1.24	n.d.
	valine	1.76	2.39
	N-acetylvaline	1.95	1.75
	3-methyl-2-oxobutyrate	1.64	2.52
	2-hydroxy-3-methylvalerate	4.95	3.83
Methionine, Cysteine, SAM and Taurine Metabolism	methionine	1.03	1.21
	N-acetylmethionine	1.24	1.28
	hypotaurine	0.35	1.55
Urea cycle; Arginine and Proline Metabolism	arginine	0.86	1.14
	proline	1.61	0.97
	N-acetylproline	2.55	n.d.
Creatine Metabolism	guanidinoacetate	2.23	2.11
	creatine	1.31	1.33
	creatinine	0.98	1.24
	creatine phosphate	0.29	n.d.
Glutathione Metabolism	glutathione, oxidized (GSSG)	n.d.	0.92

Table 4 footer: Amount was calculated as ratio (fold change) of the diabetic vs. non-diabetic value (Mean MW, *n* = 8). The statistical significance is indicated in the list by colors with *p* ≤ 0.05 in dark green and red and those approaching significance (0.05 < *p* < 0.10) with light green and red.

**Table 5:** List of selected nucleotides that were detected in maternal plasma and blastocysts cavity fluid (BF) in non-diabetic and diabetic rabbits.

Sub Pathway	Biochemical Name	Plasma	BF
Purine Metabolism, (Hypo)Xanthine/Inosine containing	inosine	0.75	0.83
	urate	1.69	2.80
	allantoin	1.00	1.76
Purine Metabolism, Adenine containing	adenosine 5’-diphosphate (ADP)	0.60	n.d.
	adenosine 5’-monophosphate (AMP)	0.93	0.49
	adenosine 3’,5’-cyclic monophosphate (cAMP)	1.24	1.21
	adenosine	1.28	0.70
	adenine	0.72	0.74
Purine Metabolism, Guanine containing	guanosine-3’,5’-cyclic monophosphate (cGMP)	1.39	1.09
	guanosine	0.94	1.13
	guanine	1.28	1.32
Pyrimidine Metabolism, Uracil containing	uridine	0.87	0.96
	uracil	1.19	0.91
	beta-alanine	1.56	1.49
Pyrimidine Metabolism, Cytidine containing	cytidine	1.09	0.90
	cytosine	1.12	1.16
Pyrimidine Metabolism, Thymine containing	thymidine	1.15	1.05
	thymine	0.94	0.98

Fold changes were calculated as diabetic vs. non-diabetic. Cells that achieved statistical significance (*p* ≤ 0.05) were indicated with dark green and red and those approaching significance (0.05 < *p* < 0.10) with light green and red.

**Table 6:** List of selected cofactors and vitamins that were detected in maternal plasma and blastocysts cavity fluid (BF) in non-diabetic and diabetic rabbits.

Sub Pathway	Biochemical Name	Plasma	BF
Nicotinate and Nicotinamide Metabolism	nicotinamide	0.91	0.80
Riboflavin Metabolism	flavin adenine dinucleotide (FAD)	0.70	n.d.
Pantothenate and CoA Metabolism	pantothenate	1.28	0.84
Tocopherol Metabolism	alpha-tocopherol	1.46	n.d.
Biotin Metabolism	biotin	n.d.	0.51
Pterin Metabolism	pterin	n.d.	0.83
Hemoglobin and Porphyrin Metabolism	bilirubin (Z,Z)	2.69	n.d.
Thiamine Metabolism	thiamin (Vitamin B1)	0.65	n.d.
Vitamin A Metabolism	retinol (Vitamin A)	1.65	n.d.
Vitamin B6 Metabolism	pyridoxamine	0.46	n.d.
	pyridoxal	1.04	0.44

Fold changes were calculated as diabetic vs. non-diabetic. Cells that achieved statistical significance (*p* ≤ 0.05) were indicated with dark green and red and those approaching significance (0.05 < *p* < 0.10) with light green and red.

**Table 7:** Parameters of blastocysts used for the metabolic analyses in blastocoel fluid by Metabolon^®^**.**

Blastocysts at Day 6 p.c.	*n*	Diameter Mean ± SD (mm)	Volume Mean (µL)	Gastrulation Stage 1/2
Non-diabetic	40	4.147 ± 0.080	26.57 ± 1.47	17/19
Diabetic	40	4.076 ± 0.126	26.03 ± 1.27	18/18
*p*-value		0.49	0.77	0.58

## Supplementary Materials

Supplementary materials can be found at https://www.mdpi.com/1422-0067/21/3/919/s1.

## Abbreviations

1,5-AG	1,5 anhydroglucitol
ADP	adenosine 5’-diphosphate
AGE	advanced glycation end products
BCAA	branched chain amino acid
BF	blastocysts cavity fluid, blastocoel fluid
BHBA	body3-hydroxybutyrate
CML	carboxymethyllysine
CPT1	carnitine palmitoyltransferase 1
d6	day 6
dpc	day post-coitum
DT1	diabetes mellitus type 1
ESI	electrospray ionization
FAD	flavin adenine dinucleotide
GDM	gestational diabetes mellitus
GLUT	Glucose transporter protein
IVF	in vitro fertilisation
NADPH	nicotinamide adenine dinucleotide phosphate
p38 MAPK	p38 mitogen-activated protein kinase
p.c.	post coitum
PPP	pentose phosphate pathway
TCA	tricarboxylic acid cycle
