# Supplementary material for: Metabolic Profiling in Blastocoel Fluid and Blood Plasma of Diabetic Rabbits
Source: Int J Mol Sci. 2020 Jan 30;21(3):919. doi: 10.3390/ijms21030919 (PMC7037143; doi:10.3390/ijms21030919)
Supplement: Supplementary file 1 [file ijms-21-00919-s001.zip › ijms-678965-supplementary.pptx]

## Slide 1
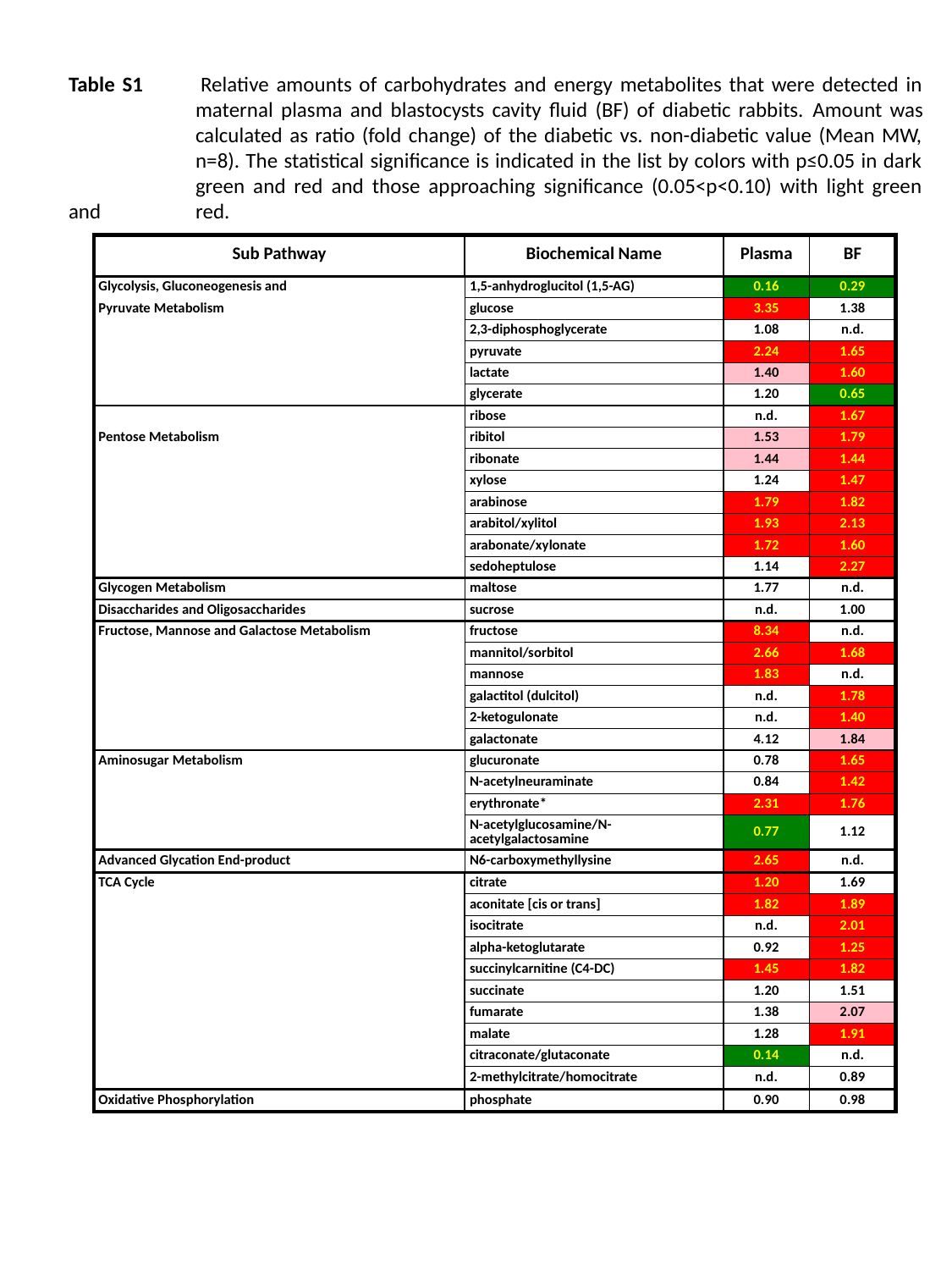

Table S1 	Relative amounts of carbohydrates and energy metabolites that were detected in 	maternal plasma and blastocysts cavity fluid (BF) of diabetic rabbits. Amount was 	calculated as ratio (fold change) of the diabetic vs. non-diabetic value (Mean MW, 	n=8). The statistical significance is indicated in the list by colors with p≤0.05 in dark 	green and red and those approaching significance (0.05<p<0.10) with light green and 	red.
| Sub Pathway | Biochemical Name | Plasma | BF |
| --- | --- | --- | --- |
| Glycolysis, Gluconeogenesis and | 1,5-anhydroglucitol (1,5-AG) | 0.16 | 0.29 |
| Pyruvate Metabolism | glucose | 3.35 | 1.38 |
| | 2,3-diphosphoglycerate | 1.08 | n.d. |
| | pyruvate | 2.24 | 1.65 |
| | lactate | 1.40 | 1.60 |
| | glycerate | 1.20 | 0.65 |
| | ribose | n.d. | 1.67 |
| Pentose Metabolism | ribitol | 1.53 | 1.79 |
| | ribonate | 1.44 | 1.44 |
| | xylose | 1.24 | 1.47 |
| | arabinose | 1.79 | 1.82 |
| | arabitol/xylitol | 1.93 | 2.13 |
| | arabonate/xylonate | 1.72 | 1.60 |
| | sedoheptulose | 1.14 | 2.27 |
| Glycogen Metabolism | maltose | 1.77 | n.d. |
| Disaccharides and Oligosaccharides | sucrose | n.d. | 1.00 |
| Fructose, Mannose and Galactose Metabolism | fructose | 8.34 | n.d. |
| | mannitol/sorbitol | 2.66 | 1.68 |
| | mannose | 1.83 | n.d. |
| | galactitol (dulcitol) | n.d. | 1.78 |
| | 2-ketogulonate | n.d. | 1.40 |
| | galactonate | 4.12 | 1.84 |
| Aminosugar Metabolism | glucuronate | 0.78 | 1.65 |
| | N-acetylneuraminate | 0.84 | 1.42 |
| | erythronate\* | 2.31 | 1.76 |
| | N-acetylglucosamine/N-acetylgalactosamine | 0.77 | 1.12 |
| Advanced Glycation End-product | N6-carboxymethyllysine | 2.65 | n.d. |
| TCA Cycle | citrate | 1.20 | 1.69 |
| | aconitate [cis or trans] | 1.82 | 1.89 |
| | isocitrate | n.d. | 2.01 |
| | alpha-ketoglutarate | 0.92 | 1.25 |
| | succinylcarnitine (C4-DC) | 1.45 | 1.82 |
| | succinate | 1.20 | 1.51 |
| | fumarate | 1.38 | 2.07 |
| | malate | 1.28 | 1.91 |
| | citraconate/glutaconate | 0.14 | n.d. |
| | 2-methylcitrate/homocitrate | n.d. | 0.89 |
| Oxidative Phosphorylation | phosphate | 0.90 | 0.98 |

## Slide 2
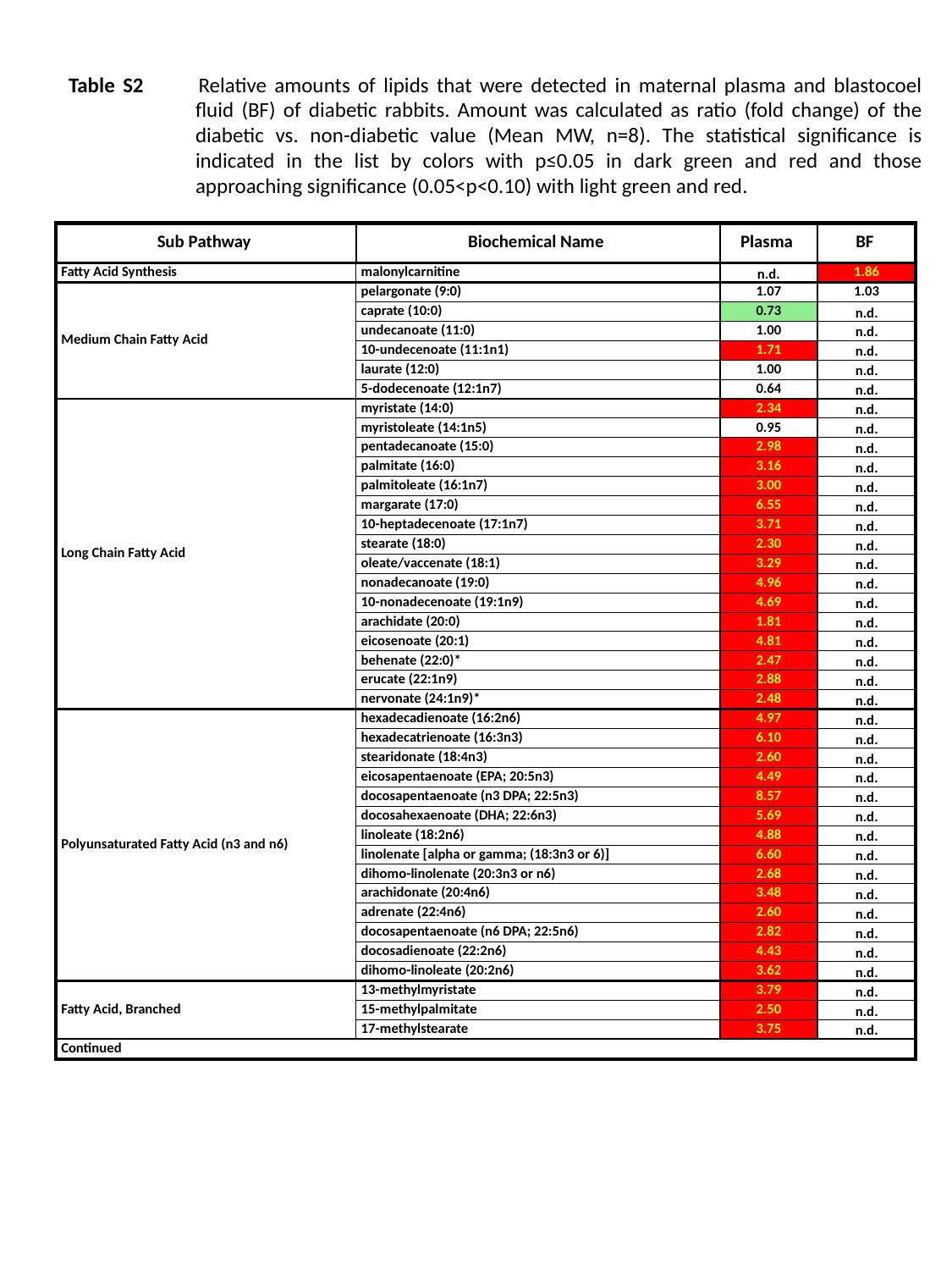

Table S2	Relative amounts of lipids that were detected in maternal plasma and blastocoel 	fluid (BF) of diabetic rabbits. Amount was calculated as ratio (fold change) of the 	diabetic vs. non-diabetic value (Mean MW, n=8). The statistical significance is 	indicated in the list by colors with p≤0.05 in dark green and red and those 	approaching significance (0.05<p<0.10) with light green and red.
| Sub Pathway | Biochemical Name | Plasma | BF |
| --- | --- | --- | --- |
| Fatty Acid Synthesis | malonylcarnitine | n.d. | 1.86 |
| Medium Chain Fatty Acid | pelargonate (9:0) | 1.07 | 1.03 |
| | caprate (10:0) | 0.73 | n.d. |
| | undecanoate (11:0) | 1.00 | n.d. |
| | 10-undecenoate (11:1n1) | 1.71 | n.d. |
| | laurate (12:0) | 1.00 | n.d. |
| | 5-dodecenoate (12:1n7) | 0.64 | n.d. |
| Long Chain Fatty Acid | myristate (14:0) | 2.34 | n.d. |
| | myristoleate (14:1n5) | 0.95 | n.d. |
| | pentadecanoate (15:0) | 2.98 | n.d. |
| | palmitate (16:0) | 3.16 | n.d. |
| | palmitoleate (16:1n7) | 3.00 | n.d. |
| | margarate (17:0) | 6.55 | n.d. |
| | 10-heptadecenoate (17:1n7) | 3.71 | n.d. |
| | stearate (18:0) | 2.30 | n.d. |
| | oleate/vaccenate (18:1) | 3.29 | n.d. |
| | nonadecanoate (19:0) | 4.96 | n.d. |
| | 10-nonadecenoate (19:1n9) | 4.69 | n.d. |
| | arachidate (20:0) | 1.81 | n.d. |
| | eicosenoate (20:1) | 4.81 | n.d. |
| | behenate (22:0)\* | 2.47 | n.d. |
| | erucate (22:1n9) | 2.88 | n.d. |
| | nervonate (24:1n9)\* | 2.48 | n.d. |
| Polyunsaturated Fatty Acid (n3 and n6) | hexadecadienoate (16:2n6) | 4.97 | n.d. |
| | hexadecatrienoate (16:3n3) | 6.10 | n.d. |
| | stearidonate (18:4n3) | 2.60 | n.d. |
| | eicosapentaenoate (EPA; 20:5n3) | 4.49 | n.d. |
| | docosapentaenoate (n3 DPA; 22:5n3) | 8.57 | n.d. |
| | docosahexaenoate (DHA; 22:6n3) | 5.69 | n.d. |
| | linoleate (18:2n6) | 4.88 | n.d. |
| | linolenate [alpha or gamma; (18:3n3 or 6)] | 6.60 | n.d. |
| | dihomo-linolenate (20:3n3 or n6) | 2.68 | n.d. |
| | arachidonate (20:4n6) | 3.48 | n.d. |
| | adrenate (22:4n6) | 2.60 | n.d. |
| | docosapentaenoate (n6 DPA; 22:5n6) | 2.82 | n.d. |
| | docosadienoate (22:2n6) | 4.43 | n.d. |
| | dihomo-linoleate (20:2n6) | 3.62 | n.d. |
| Fatty Acid, Branched | 13-methylmyristate | 3.79 | n.d. |
| | 15-methylpalmitate | 2.50 | n.d. |
| | 17-methylstearate | 3.75 | n.d. |
| Continued | | | |

## Slide 3
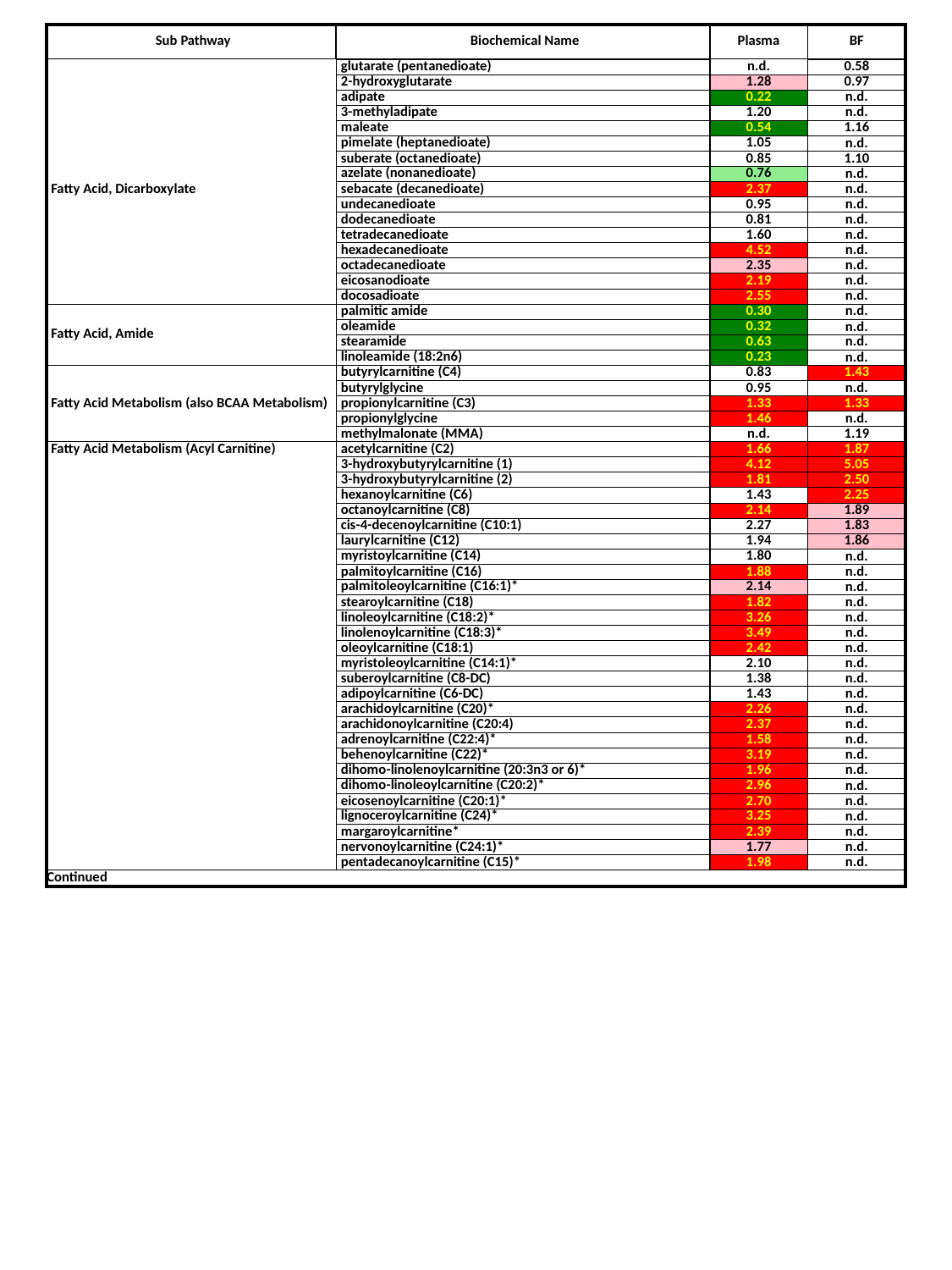

| Sub Pathway | Biochemical Name | Plasma | BF |
| --- | --- | --- | --- |
| | glutarate (pentanedioate) | n.d. | 0.58 |
| Fatty Acid, Dicarboxylate | 2-hydroxyglutarate | 1.28 | 0.97 |
| | adipate | 0.22 | n.d. |
| | 3-methyladipate | 1.20 | n.d. |
| | maleate | 0.54 | 1.16 |
| | pimelate (heptanedioate) | 1.05 | n.d. |
| | suberate (octanedioate) | 0.85 | 1.10 |
| | azelate (nonanedioate) | 0.76 | n.d. |
| | sebacate (decanedioate) | 2.37 | n.d. |
| | undecanedioate | 0.95 | n.d. |
| | dodecanedioate | 0.81 | n.d. |
| | tetradecanedioate | 1.60 | n.d. |
| | hexadecanedioate | 4.52 | n.d. |
| | octadecanedioate | 2.35 | n.d. |
| | eicosanodioate | 2.19 | n.d. |
| | docosadioate | 2.55 | n.d. |
| Fatty Acid, Amide | palmitic amide | 0.30 | n.d. |
| | oleamide | 0.32 | n.d. |
| | stearamide | 0.63 | n.d. |
| | linoleamide (18:2n6) | 0.23 | n.d. |
| Fatty Acid Metabolism (also BCAA Metabolism) | butyrylcarnitine (C4) | 0.83 | 1.43 |
| | butyrylglycine | 0.95 | n.d. |
| | propionylcarnitine (C3) | 1.33 | 1.33 |
| | propionylglycine | 1.46 | n.d. |
| | methylmalonate (MMA) | n.d. | 1.19 |
| Fatty Acid Metabolism (Acyl Carnitine) | acetylcarnitine (C2) | 1.66 | 1.87 |
| | 3-hydroxybutyrylcarnitine (1) | 4.12 | 5.05 |
| | 3-hydroxybutyrylcarnitine (2) | 1.81 | 2.50 |
| | hexanoylcarnitine (C6) | 1.43 | 2.25 |
| | octanoylcarnitine (C8) | 2.14 | 1.89 |
| | cis-4-decenoylcarnitine (C10:1) | 2.27 | 1.83 |
| | laurylcarnitine (C12) | 1.94 | 1.86 |
| | myristoylcarnitine (C14) | 1.80 | n.d. |
| | palmitoylcarnitine (C16) | 1.88 | n.d. |
| | palmitoleoylcarnitine (C16:1)\* | 2.14 | n.d. |
| | stearoylcarnitine (C18) | 1.82 | n.d. |
| | linoleoylcarnitine (C18:2)\* | 3.26 | n.d. |
| | linolenoylcarnitine (C18:3)\* | 3.49 | n.d. |
| | oleoylcarnitine (C18:1) | 2.42 | n.d. |
| | myristoleoylcarnitine (C14:1)\* | 2.10 | n.d. |
| | suberoylcarnitine (C8-DC) | 1.38 | n.d. |
| | adipoylcarnitine (C6-DC) | 1.43 | n.d. |
| | arachidoylcarnitine (C20)\* | 2.26 | n.d. |
| | arachidonoylcarnitine (C20:4) | 2.37 | n.d. |
| | adrenoylcarnitine (C22:4)\* | 1.58 | n.d. |
| | behenoylcarnitine (C22)\* | 3.19 | n.d. |
| | dihomo-linolenoylcarnitine (20:3n3 or 6)\* | 1.96 | n.d. |
| | dihomo-linoleoylcarnitine (C20:2)\* | 2.96 | n.d. |
| | eicosenoylcarnitine (C20:1)\* | 2.70 | n.d. |
| | lignoceroylcarnitine (C24)\* | 3.25 | n.d. |
| | margaroylcarnitine\* | 2.39 | n.d. |
| | nervonoylcarnitine (C24:1)\* | 1.77 | n.d. |
| | pentadecanoylcarnitine (C15)\* | 1.98 | n.d. |
| Continued | | | |

## Slide 4
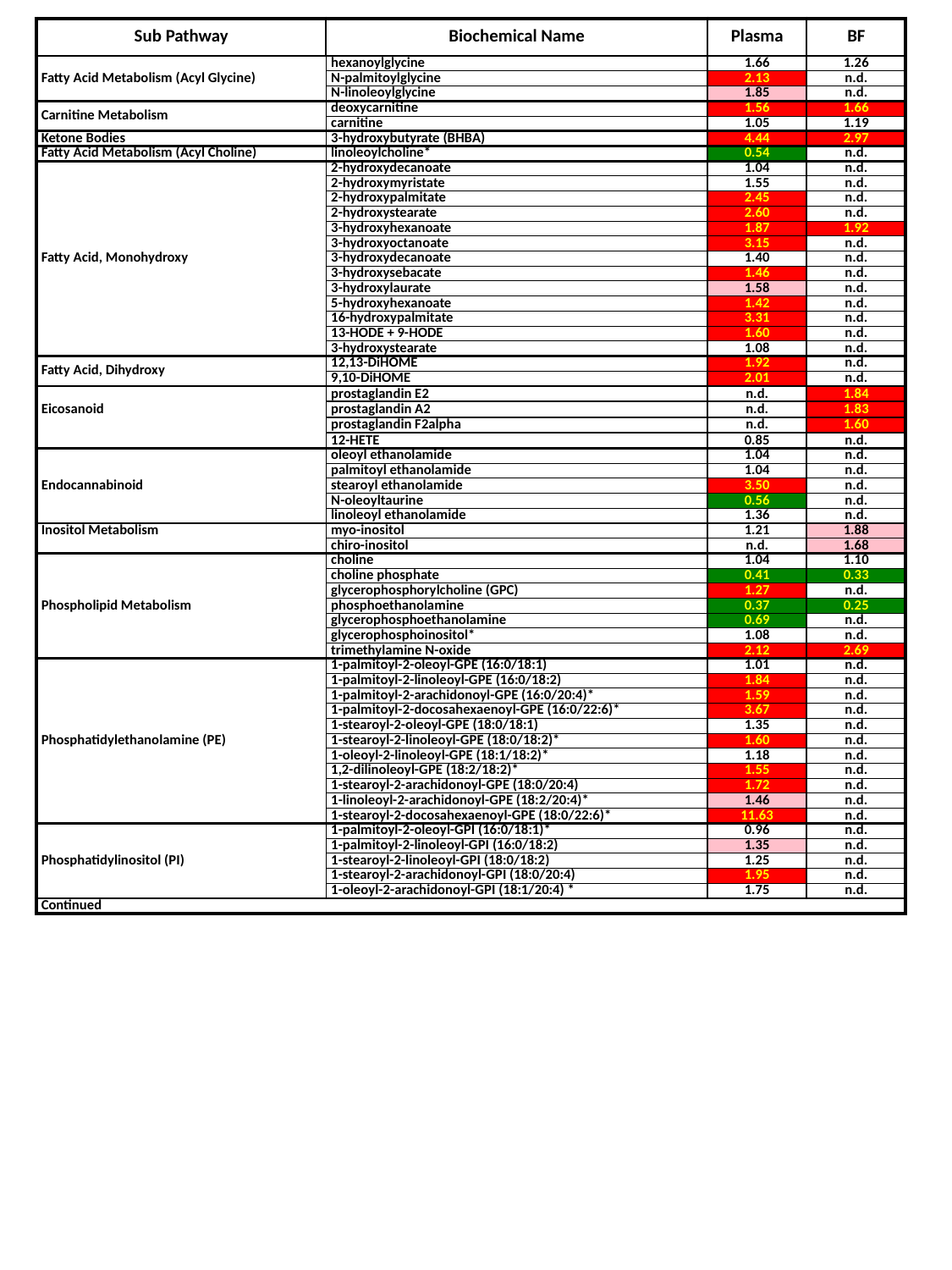

| Sub Pathway | Biochemical Name | Plasma | BF |
| --- | --- | --- | --- |
| Fatty Acid Metabolism (Acyl Glycine) | hexanoylglycine | 1.66 | 1.26 |
| | N-palmitoylglycine | 2.13 | n.d. |
| | N-linoleoylglycine | 1.85 | n.d. |
| Carnitine Metabolism | deoxycarnitine | 1.56 | 1.66 |
| | carnitine | 1.05 | 1.19 |
| Ketone Bodies | 3-hydroxybutyrate (BHBA) | 4.44 | 2.97 |
| Fatty Acid Metabolism (Acyl Choline) | linoleoylcholine\* | 0.54 | n.d. |
| Fatty Acid, Monohydroxy | 2-hydroxydecanoate | 1.04 | n.d. |
| | 2-hydroxymyristate | 1.55 | n.d. |
| | 2-hydroxypalmitate | 2.45 | n.d. |
| | 2-hydroxystearate | 2.60 | n.d. |
| | 3-hydroxyhexanoate | 1.87 | 1.92 |
| | 3-hydroxyoctanoate | 3.15 | n.d. |
| | 3-hydroxydecanoate | 1.40 | n.d. |
| | 3-hydroxysebacate | 1.46 | n.d. |
| | 3-hydroxylaurate | 1.58 | n.d. |
| | 5-hydroxyhexanoate | 1.42 | n.d. |
| | 16-hydroxypalmitate | 3.31 | n.d. |
| | 13-HODE + 9-HODE | 1.60 | n.d. |
| | 3-hydroxystearate | 1.08 | n.d. |
| Fatty Acid, Dihydroxy | 12,13-DiHOME | 1.92 | n.d. |
| | 9,10-DiHOME | 2.01 | n.d. |
| | prostaglandin E2 | n.d. | 1.84 |
| Eicosanoid | prostaglandin A2 | n.d. | 1.83 |
| | prostaglandin F2alpha | n.d. | 1.60 |
| | 12-HETE | 0.85 | n.d. |
| Endocannabinoid | oleoyl ethanolamide | 1.04 | n.d. |
| | palmitoyl ethanolamide | 1.04 | n.d. |
| | stearoyl ethanolamide | 3.50 | n.d. |
| | N-oleoyltaurine | 0.56 | n.d. |
| | linoleoyl ethanolamide | 1.36 | n.d. |
| Inositol Metabolism | myo-inositol | 1.21 | 1.88 |
| | chiro-inositol | n.d. | 1.68 |
| Phospholipid Metabolism | choline | 1.04 | 1.10 |
| | choline phosphate | 0.41 | 0.33 |
| | glycerophosphorylcholine (GPC) | 1.27 | n.d. |
| | phosphoethanolamine | 0.37 | 0.25 |
| | glycerophosphoethanolamine | 0.69 | n.d. |
| | glycerophosphoinositol\* | 1.08 | n.d. |
| | trimethylamine N-oxide | 2.12 | 2.69 |
| Phosphatidylethanolamine (PE) | 1-palmitoyl-2-oleoyl-GPE (16:0/18:1) | 1.01 | n.d. |
| | 1-palmitoyl-2-linoleoyl-GPE (16:0/18:2) | 1.84 | n.d. |
| | 1-palmitoyl-2-arachidonoyl-GPE (16:0/20:4)\* | 1.59 | n.d. |
| | 1-palmitoyl-2-docosahexaenoyl-GPE (16:0/22:6)\* | 3.67 | n.d. |
| | 1-stearoyl-2-oleoyl-GPE (18:0/18:1) | 1.35 | n.d. |
| | 1-stearoyl-2-linoleoyl-GPE (18:0/18:2)\* | 1.60 | n.d. |
| | 1-oleoyl-2-linoleoyl-GPE (18:1/18:2)\* | 1.18 | n.d. |
| | 1,2-dilinoleoyl-GPE (18:2/18:2)\* | 1.55 | n.d. |
| | 1-stearoyl-2-arachidonoyl-GPE (18:0/20:4) | 1.72 | n.d. |
| | 1-linoleoyl-2-arachidonoyl-GPE (18:2/20:4)\* | 1.46 | n.d. |
| | 1-stearoyl-2-docosahexaenoyl-GPE (18:0/22:6)\* | 11.63 | n.d. |
| Phosphatidylinositol (PI) | 1-palmitoyl-2-oleoyl-GPI (16:0/18:1)\* | 0.96 | n.d. |
| | 1-palmitoyl-2-linoleoyl-GPI (16:0/18:2) | 1.35 | n.d. |
| | 1-stearoyl-2-linoleoyl-GPI (18:0/18:2) | 1.25 | n.d. |
| | 1-stearoyl-2-arachidonoyl-GPI (18:0/20:4) | 1.95 | n.d. |
| | 1-oleoyl-2-arachidonoyl-GPI (18:1/20:4) \* | 1.75 | n.d. |
| Continued | | | |

## Slide 5
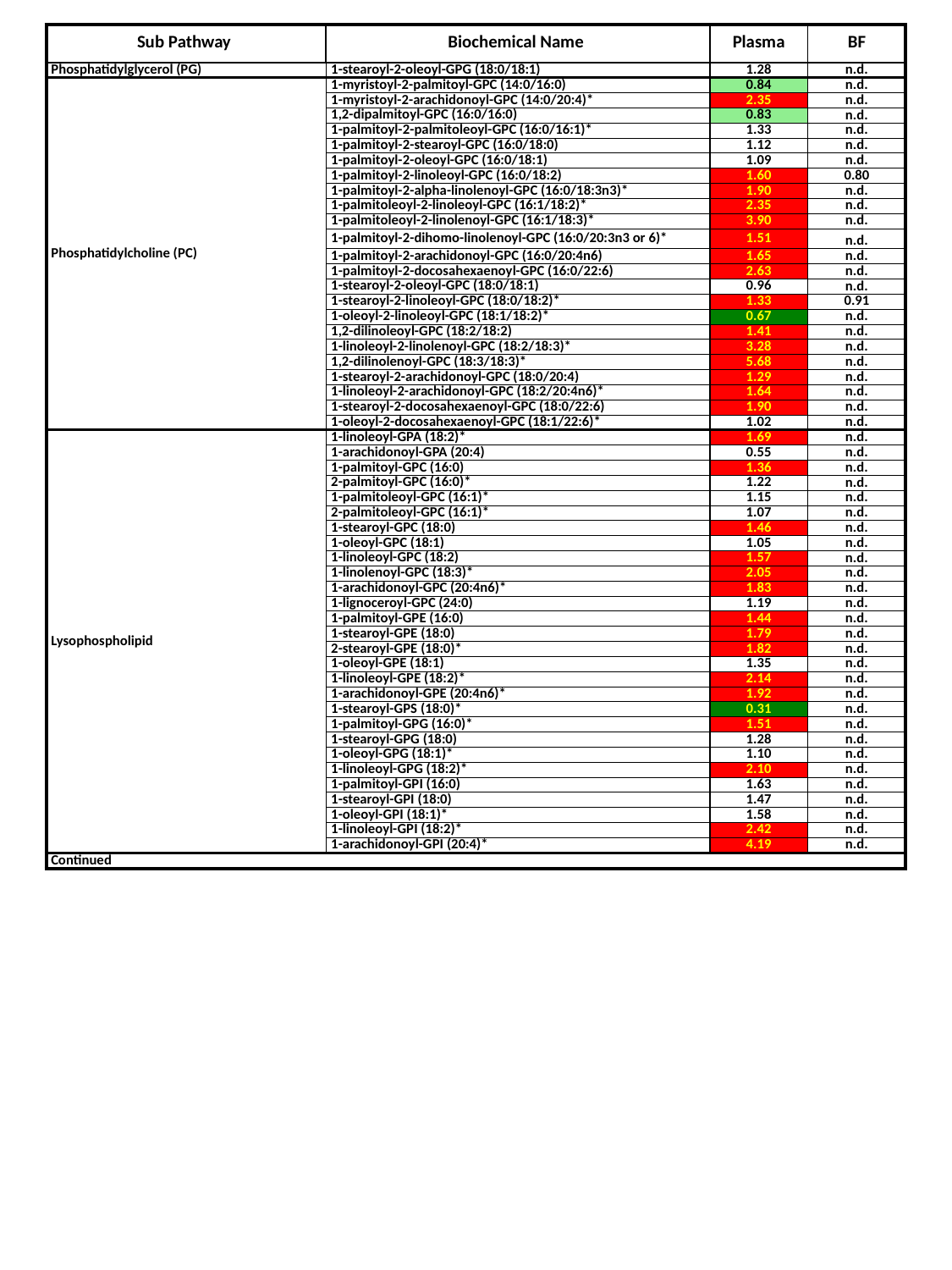

| Sub Pathway | Biochemical Name | Plasma | BF |
| --- | --- | --- | --- |
| Phosphatidylglycerol (PG) | 1-stearoyl-2-oleoyl-GPG (18:0/18:1) | 1.28 | n.d. |
| Phosphatidylcholine (PC) | 1-myristoyl-2-palmitoyl-GPC (14:0/16:0) | 0.84 | n.d. |
| | 1-myristoyl-2-arachidonoyl-GPC (14:0/20:4)\* | 2.35 | n.d. |
| | 1,2-dipalmitoyl-GPC (16:0/16:0) | 0.83 | n.d. |
| | 1-palmitoyl-2-palmitoleoyl-GPC (16:0/16:1)\* | 1.33 | n.d. |
| | 1-palmitoyl-2-stearoyl-GPC (16:0/18:0) | 1.12 | n.d. |
| | 1-palmitoyl-2-oleoyl-GPC (16:0/18:1) | 1.09 | n.d. |
| | 1-palmitoyl-2-linoleoyl-GPC (16:0/18:2) | 1.60 | 0.80 |
| | 1-palmitoyl-2-alpha-linolenoyl-GPC (16:0/18:3n3)\* | 1.90 | n.d. |
| | 1-palmitoleoyl-2-linoleoyl-GPC (16:1/18:2)\* | 2.35 | n.d. |
| | 1-palmitoleoyl-2-linolenoyl-GPC (16:1/18:3)\* | 3.90 | n.d. |
| | 1-palmitoyl-2-dihomo-linolenoyl-GPC (16:0/20:3n3 or 6)\* | 1.51 | n.d. |
| | 1-palmitoyl-2-arachidonoyl-GPC (16:0/20:4n6) | 1.65 | n.d. |
| | 1-palmitoyl-2-docosahexaenoyl-GPC (16:0/22:6) | 2.63 | n.d. |
| | 1-stearoyl-2-oleoyl-GPC (18:0/18:1) | 0.96 | n.d. |
| | 1-stearoyl-2-linoleoyl-GPC (18:0/18:2)\* | 1.33 | 0.91 |
| | 1-oleoyl-2-linoleoyl-GPC (18:1/18:2)\* | 0.67 | n.d. |
| | 1,2-dilinoleoyl-GPC (18:2/18:2) | 1.41 | n.d. |
| | 1-linoleoyl-2-linolenoyl-GPC (18:2/18:3)\* | 3.28 | n.d. |
| | 1,2-dilinolenoyl-GPC (18:3/18:3)\* | 5.68 | n.d. |
| | 1-stearoyl-2-arachidonoyl-GPC (18:0/20:4) | 1.29 | n.d. |
| | 1-linoleoyl-2-arachidonoyl-GPC (18:2/20:4n6)\* | 1.64 | n.d. |
| | 1-stearoyl-2-docosahexaenoyl-GPC (18:0/22:6) | 1.90 | n.d. |
| | 1-oleoyl-2-docosahexaenoyl-GPC (18:1/22:6)\* | 1.02 | n.d. |
| Lysophospholipid | 1-linoleoyl-GPA (18:2)\* | 1.69 | n.d. |
| | 1-arachidonoyl-GPA (20:4) | 0.55 | n.d. |
| | 1-palmitoyl-GPC (16:0) | 1.36 | n.d. |
| | 2-palmitoyl-GPC (16:0)\* | 1.22 | n.d. |
| | 1-palmitoleoyl-GPC (16:1)\* | 1.15 | n.d. |
| | 2-palmitoleoyl-GPC (16:1)\* | 1.07 | n.d. |
| | 1-stearoyl-GPC (18:0) | 1.46 | n.d. |
| | 1-oleoyl-GPC (18:1) | 1.05 | n.d. |
| | 1-linoleoyl-GPC (18:2) | 1.57 | n.d. |
| | 1-linolenoyl-GPC (18:3)\* | 2.05 | n.d. |
| | 1-arachidonoyl-GPC (20:4n6)\* | 1.83 | n.d. |
| | 1-lignoceroyl-GPC (24:0) | 1.19 | n.d. |
| | 1-palmitoyl-GPE (16:0) | 1.44 | n.d. |
| | 1-stearoyl-GPE (18:0) | 1.79 | n.d. |
| | 2-stearoyl-GPE (18:0)\* | 1.82 | n.d. |
| | 1-oleoyl-GPE (18:1) | 1.35 | n.d. |
| | 1-linoleoyl-GPE (18:2)\* | 2.14 | n.d. |
| | 1-arachidonoyl-GPE (20:4n6)\* | 1.92 | n.d. |
| | 1-stearoyl-GPS (18:0)\* | 0.31 | n.d. |
| | 1-palmitoyl-GPG (16:0)\* | 1.51 | n.d. |
| | 1-stearoyl-GPG (18:0) | 1.28 | n.d. |
| | 1-oleoyl-GPG (18:1)\* | 1.10 | n.d. |
| | 1-linoleoyl-GPG (18:2)\* | 2.10 | n.d. |
| | 1-palmitoyl-GPI (16:0) | 1.63 | n.d. |
| | 1-stearoyl-GPI (18:0) | 1.47 | n.d. |
| | 1-oleoyl-GPI (18:1)\* | 1.58 | n.d. |
| | 1-linoleoyl-GPI (18:2)\* | 2.42 | n.d. |
| | 1-arachidonoyl-GPI (20:4)\* | 4.19 | n.d. |
| Continued | | | |

## Slide 6
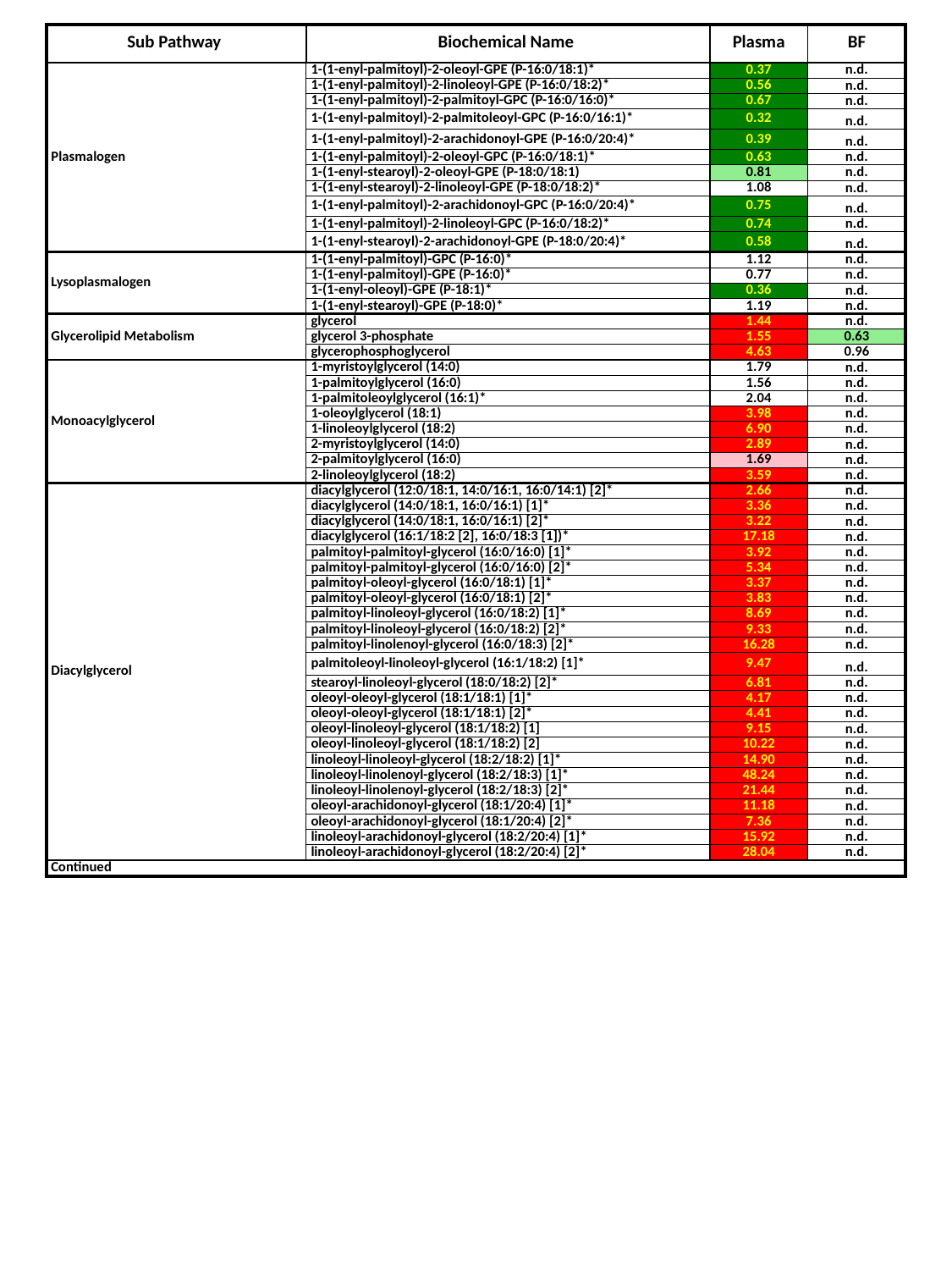

| Sub Pathway | Biochemical Name | Plasma | BF |
| --- | --- | --- | --- |
| Plasmalogen | 1-(1-enyl-palmitoyl)-2-oleoyl-GPE (P-16:0/18:1)\* | 0.37 | n.d. |
| | 1-(1-enyl-palmitoyl)-2-linoleoyl-GPE (P-16:0/18:2)\* | 0.56 | n.d. |
| | 1-(1-enyl-palmitoyl)-2-palmitoyl-GPC (P-16:0/16:0)\* | 0.67 | n.d. |
| | 1-(1-enyl-palmitoyl)-2-palmitoleoyl-GPC (P-16:0/16:1)\* | 0.32 | n.d. |
| | 1-(1-enyl-palmitoyl)-2-arachidonoyl-GPE (P-16:0/20:4)\* | 0.39 | n.d. |
| | 1-(1-enyl-palmitoyl)-2-oleoyl-GPC (P-16:0/18:1)\* | 0.63 | n.d. |
| | 1-(1-enyl-stearoyl)-2-oleoyl-GPE (P-18:0/18:1) | 0.81 | n.d. |
| | 1-(1-enyl-stearoyl)-2-linoleoyl-GPE (P-18:0/18:2)\* | 1.08 | n.d. |
| | 1-(1-enyl-palmitoyl)-2-arachidonoyl-GPC (P-16:0/20:4)\* | 0.75 | n.d. |
| | 1-(1-enyl-palmitoyl)-2-linoleoyl-GPC (P-16:0/18:2)\* | 0.74 | n.d. |
| | 1-(1-enyl-stearoyl)-2-arachidonoyl-GPE (P-18:0/20:4)\* | 0.58 | n.d. |
| Lysoplasmalogen | 1-(1-enyl-palmitoyl)-GPC (P-16:0)\* | 1.12 | n.d. |
| | 1-(1-enyl-palmitoyl)-GPE (P-16:0)\* | 0.77 | n.d. |
| | 1-(1-enyl-oleoyl)-GPE (P-18:1)\* | 0.36 | n.d. |
| | 1-(1-enyl-stearoyl)-GPE (P-18:0)\* | 1.19 | n.d. |
| Glycerolipid Metabolism | glycerol | 1.44 | n.d. |
| | glycerol 3-phosphate | 1.55 | 0.63 |
| | glycerophosphoglycerol | 4.63 | 0.96 |
| Monoacylglycerol | 1-myristoylglycerol (14:0) | 1.79 | n.d. |
| | 1-palmitoylglycerol (16:0) | 1.56 | n.d. |
| | 1-palmitoleoylglycerol (16:1)\* | 2.04 | n.d. |
| | 1-oleoylglycerol (18:1) | 3.98 | n.d. |
| | 1-linoleoylglycerol (18:2) | 6.90 | n.d. |
| | 2-myristoylglycerol (14:0) | 2.89 | n.d. |
| | 2-palmitoylglycerol (16:0) | 1.69 | n.d. |
| | 2-linoleoylglycerol (18:2) | 3.59 | n.d. |
| Diacylglycerol | diacylglycerol (12:0/18:1, 14:0/16:1, 16:0/14:1) [2]\* | 2.66 | n.d. |
| | diacylglycerol (14:0/18:1, 16:0/16:1) [1]\* | 3.36 | n.d. |
| | diacylglycerol (14:0/18:1, 16:0/16:1) [2]\* | 3.22 | n.d. |
| | diacylglycerol (16:1/18:2 [2], 16:0/18:3 [1])\* | 17.18 | n.d. |
| | palmitoyl-palmitoyl-glycerol (16:0/16:0) [1]\* | 3.92 | n.d. |
| | palmitoyl-palmitoyl-glycerol (16:0/16:0) [2]\* | 5.34 | n.d. |
| | palmitoyl-oleoyl-glycerol (16:0/18:1) [1]\* | 3.37 | n.d. |
| | palmitoyl-oleoyl-glycerol (16:0/18:1) [2]\* | 3.83 | n.d. |
| | palmitoyl-linoleoyl-glycerol (16:0/18:2) [1]\* | 8.69 | n.d. |
| | palmitoyl-linoleoyl-glycerol (16:0/18:2) [2]\* | 9.33 | n.d. |
| | palmitoyl-linolenoyl-glycerol (16:0/18:3) [2]\* | 16.28 | n.d. |
| | palmitoleoyl-linoleoyl-glycerol (16:1/18:2) [1]\* | 9.47 | n.d. |
| | stearoyl-linoleoyl-glycerol (18:0/18:2) [2]\* | 6.81 | n.d. |
| | oleoyl-oleoyl-glycerol (18:1/18:1) [1]\* | 4.17 | n.d. |
| | oleoyl-oleoyl-glycerol (18:1/18:1) [2]\* | 4.41 | n.d. |
| | oleoyl-linoleoyl-glycerol (18:1/18:2) [1] | 9.15 | n.d. |
| | oleoyl-linoleoyl-glycerol (18:1/18:2) [2] | 10.22 | n.d. |
| | linoleoyl-linoleoyl-glycerol (18:2/18:2) [1]\* | 14.90 | n.d. |
| | linoleoyl-linolenoyl-glycerol (18:2/18:3) [1]\* | 48.24 | n.d. |
| | linoleoyl-linolenoyl-glycerol (18:2/18:3) [2]\* | 21.44 | n.d. |
| | oleoyl-arachidonoyl-glycerol (18:1/20:4) [1]\* | 11.18 | n.d. |
| | oleoyl-arachidonoyl-glycerol (18:1/20:4) [2]\* | 7.36 | n.d. |
| | linoleoyl-arachidonoyl-glycerol (18:2/20:4) [1]\* | 15.92 | n.d. |
| | linoleoyl-arachidonoyl-glycerol (18:2/20:4) [2]\* | 28.04 | n.d. |
| Continued | | | |

## Slide 7
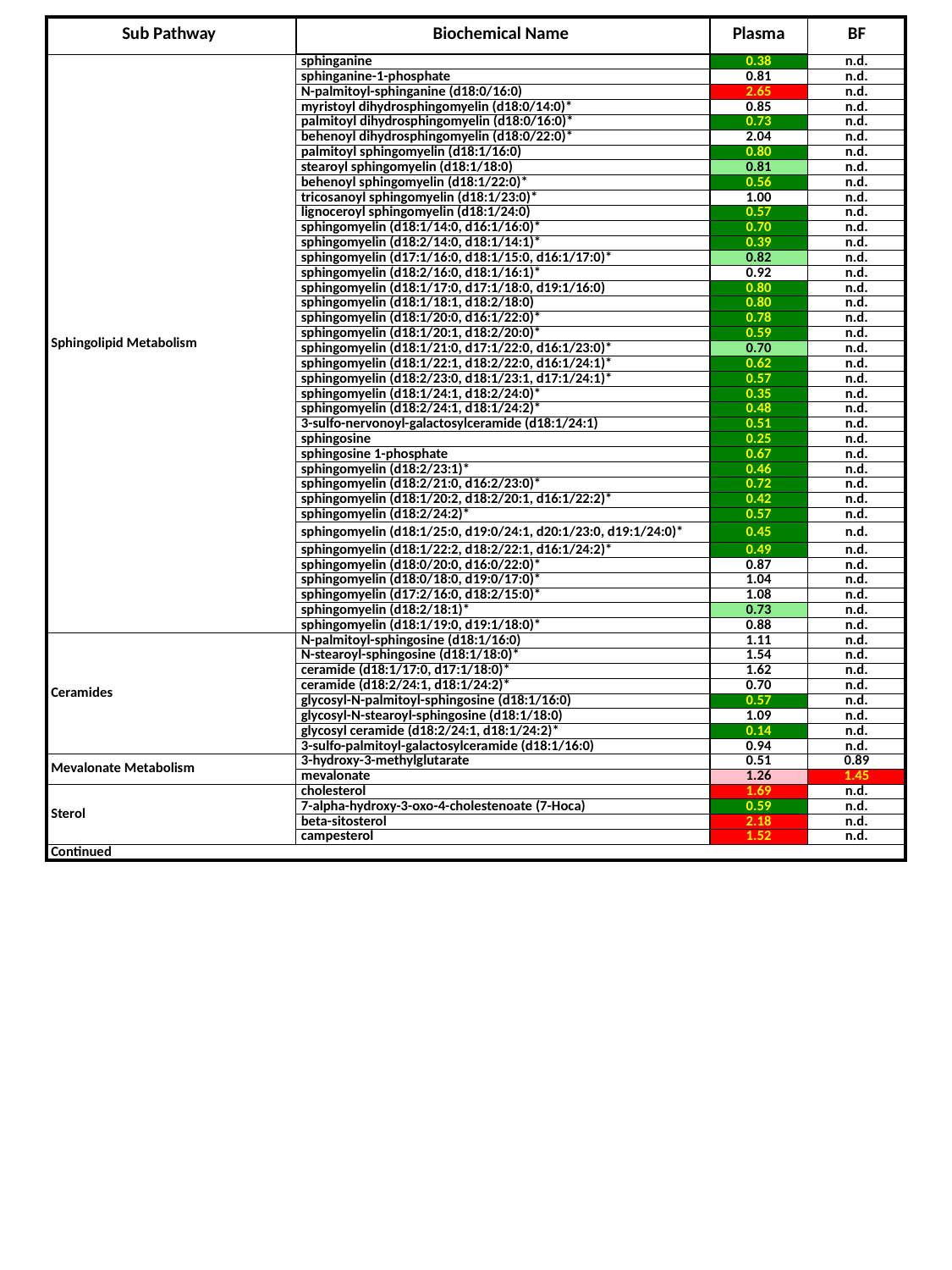

| Sub Pathway | Biochemical Name | Plasma | BF |
| --- | --- | --- | --- |
| Sphingolipid Metabolism | sphinganine | 0.38 | n.d. |
| | sphinganine-1-phosphate | 0.81 | n.d. |
| | N-palmitoyl-sphinganine (d18:0/16:0) | 2.65 | n.d. |
| | myristoyl dihydrosphingomyelin (d18:0/14:0)\* | 0.85 | n.d. |
| | palmitoyl dihydrosphingomyelin (d18:0/16:0)\* | 0.73 | n.d. |
| | behenoyl dihydrosphingomyelin (d18:0/22:0)\* | 2.04 | n.d. |
| | palmitoyl sphingomyelin (d18:1/16:0) | 0.80 | n.d. |
| | stearoyl sphingomyelin (d18:1/18:0) | 0.81 | n.d. |
| | behenoyl sphingomyelin (d18:1/22:0)\* | 0.56 | n.d. |
| | tricosanoyl sphingomyelin (d18:1/23:0)\* | 1.00 | n.d. |
| | lignoceroyl sphingomyelin (d18:1/24:0) | 0.57 | n.d. |
| | sphingomyelin (d18:1/14:0, d16:1/16:0)\* | 0.70 | n.d. |
| | sphingomyelin (d18:2/14:0, d18:1/14:1)\* | 0.39 | n.d. |
| | sphingomyelin (d17:1/16:0, d18:1/15:0, d16:1/17:0)\* | 0.82 | n.d. |
| | sphingomyelin (d18:2/16:0, d18:1/16:1)\* | 0.92 | n.d. |
| | sphingomyelin (d18:1/17:0, d17:1/18:0, d19:1/16:0) | 0.80 | n.d. |
| | sphingomyelin (d18:1/18:1, d18:2/18:0) | 0.80 | n.d. |
| | sphingomyelin (d18:1/20:0, d16:1/22:0)\* | 0.78 | n.d. |
| | sphingomyelin (d18:1/20:1, d18:2/20:0)\* | 0.59 | n.d. |
| | sphingomyelin (d18:1/21:0, d17:1/22:0, d16:1/23:0)\* | 0.70 | n.d. |
| | sphingomyelin (d18:1/22:1, d18:2/22:0, d16:1/24:1)\* | 0.62 | n.d. |
| | sphingomyelin (d18:2/23:0, d18:1/23:1, d17:1/24:1)\* | 0.57 | n.d. |
| | sphingomyelin (d18:1/24:1, d18:2/24:0)\* | 0.35 | n.d. |
| | sphingomyelin (d18:2/24:1, d18:1/24:2)\* | 0.48 | n.d. |
| | 3-sulfo-nervonoyl-galactosylceramide (d18:1/24:1) | 0.51 | n.d. |
| | sphingosine | 0.25 | n.d. |
| | sphingosine 1-phosphate | 0.67 | n.d. |
| | sphingomyelin (d18:2/23:1)\* | 0.46 | n.d. |
| | sphingomyelin (d18:2/21:0, d16:2/23:0)\* | 0.72 | n.d. |
| | sphingomyelin (d18:1/20:2, d18:2/20:1, d16:1/22:2)\* | 0.42 | n.d. |
| | sphingomyelin (d18:2/24:2)\* | 0.57 | n.d. |
| | sphingomyelin (d18:1/25:0, d19:0/24:1, d20:1/23:0, d19:1/24:0)\* | 0.45 | n.d. |
| | sphingomyelin (d18:1/22:2, d18:2/22:1, d16:1/24:2)\* | 0.49 | n.d. |
| | sphingomyelin (d18:0/20:0, d16:0/22:0)\* | 0.87 | n.d. |
| | sphingomyelin (d18:0/18:0, d19:0/17:0)\* | 1.04 | n.d. |
| | sphingomyelin (d17:2/16:0, d18:2/15:0)\* | 1.08 | n.d. |
| | sphingomyelin (d18:2/18:1)\* | 0.73 | n.d. |
| | sphingomyelin (d18:1/19:0, d19:1/18:0)\* | 0.88 | n.d. |
| Ceramides | N-palmitoyl-sphingosine (d18:1/16:0) | 1.11 | n.d. |
| | N-stearoyl-sphingosine (d18:1/18:0)\* | 1.54 | n.d. |
| | ceramide (d18:1/17:0, d17:1/18:0)\* | 1.62 | n.d. |
| | ceramide (d18:2/24:1, d18:1/24:2)\* | 0.70 | n.d. |
| | glycosyl-N-palmitoyl-sphingosine (d18:1/16:0) | 0.57 | n.d. |
| | glycosyl-N-stearoyl-sphingosine (d18:1/18:0) | 1.09 | n.d. |
| | glycosyl ceramide (d18:2/24:1, d18:1/24:2)\* | 0.14 | n.d. |
| | 3-sulfo-palmitoyl-galactosylceramide (d18:1/16:0) | 0.94 | n.d. |
| Mevalonate Metabolism | 3-hydroxy-3-methylglutarate | 0.51 | 0.89 |
| | mevalonate | 1.26 | 1.45 |
| Sterol | cholesterol | 1.69 | n.d. |
| | 7-alpha-hydroxy-3-oxo-4-cholestenoate (7-Hoca) | 0.59 | n.d. |
| | beta-sitosterol | 2.18 | n.d. |
| | campesterol | 1.52 | n.d. |
| Continued | | | |

## Slide 8
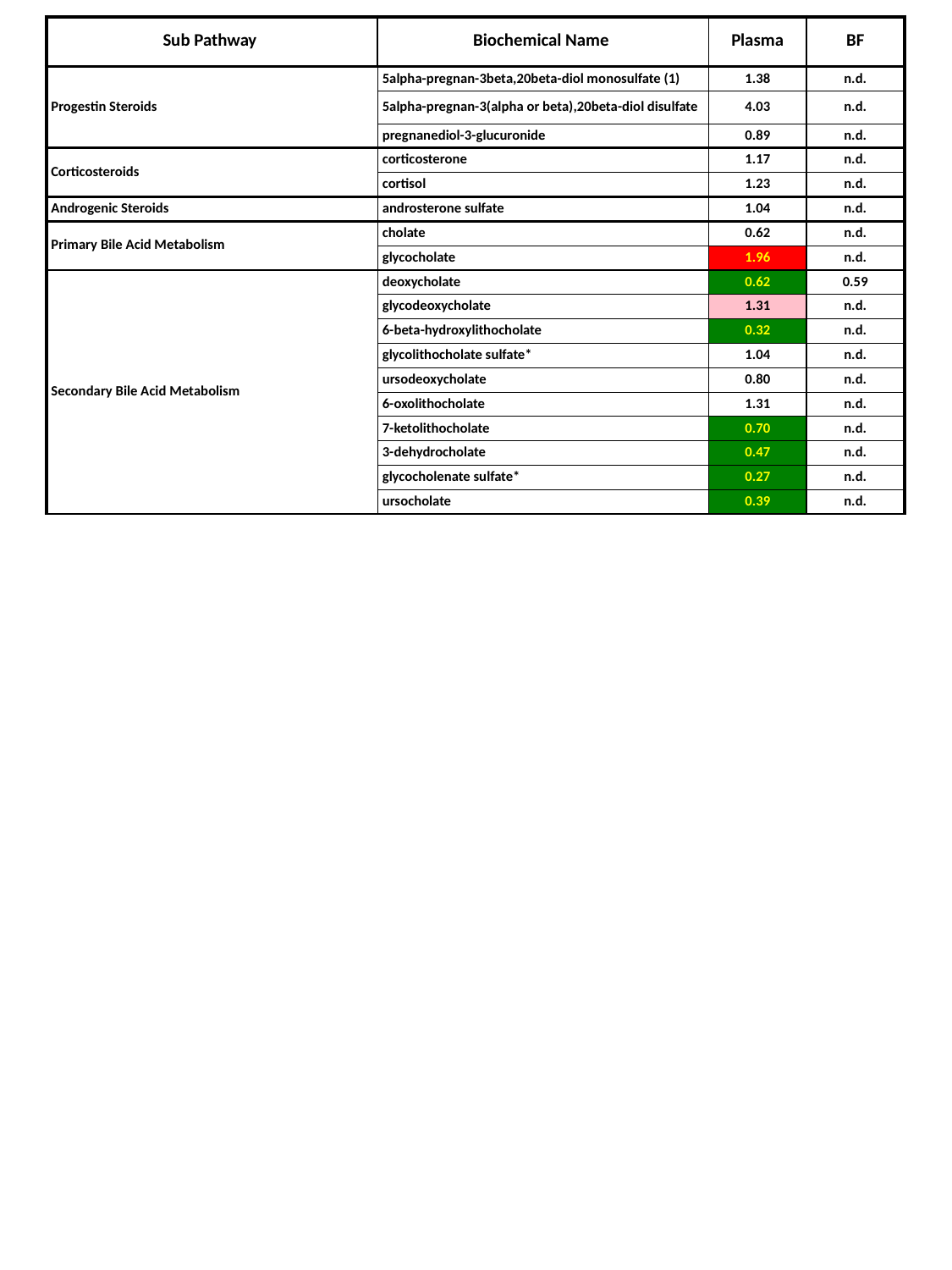

| Sub Pathway | Biochemical Name | Plasma | BF |
| --- | --- | --- | --- |
| Progestin Steroids | 5alpha-pregnan-3beta,20beta-diol monosulfate (1) | 1.38 | n.d. |
| | 5alpha-pregnan-3(alpha or beta),20beta-diol disulfate | 4.03 | n.d. |
| | pregnanediol-3-glucuronide | 0.89 | n.d. |
| Corticosteroids | corticosterone | 1.17 | n.d. |
| | cortisol | 1.23 | n.d. |
| Androgenic Steroids | androsterone sulfate | 1.04 | n.d. |
| Primary Bile Acid Metabolism | cholate | 0.62 | n.d. |
| | glycocholate | 1.96 | n.d. |
| Secondary Bile Acid Metabolism | deoxycholate | 0.62 | 0.59 |
| | glycodeoxycholate | 1.31 | n.d. |
| | 6-beta-hydroxylithocholate | 0.32 | n.d. |
| | glycolithocholate sulfate\* | 1.04 | n.d. |
| | ursodeoxycholate | 0.80 | n.d. |
| | 6-oxolithocholate | 1.31 | n.d. |
| | 7-ketolithocholate | 0.70 | n.d. |
| | 3-dehydrocholate | 0.47 | n.d. |
| | glycocholenate sulfate\* | 0.27 | n.d. |
| | ursocholate | 0.39 | n.d. |

## Slide 9
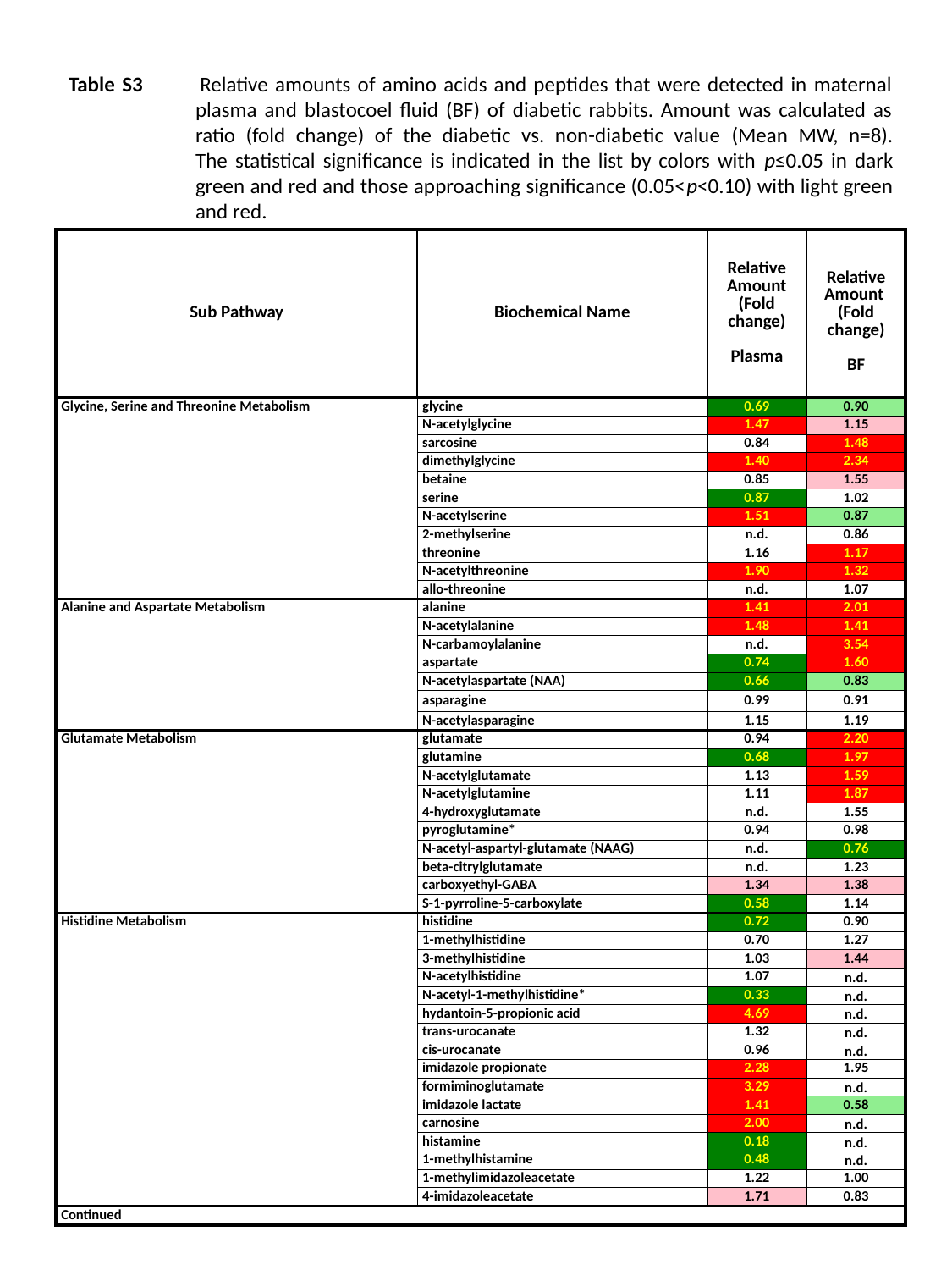

Table S3 	Relative amounts of amino acids and peptides that were detected in maternal 	plasma and blastocoel fluid (BF) of diabetic rabbits. Amount was calculated as 	ratio (fold change) of the diabetic vs. non-diabetic value (Mean MW, n=8). 	The statistical significance is indicated in the list by colors with p≤0.05 in dark 	green and red and those approaching significance (0.05<p<0.10) with light green 	and red.
| Sub Pathway | Biochemical Name | Relative Amount (Fold change) Plasma | Relative Amount (Fold change) BF |
| --- | --- | --- | --- |
| Glycine, Serine and Threonine Metabolism | glycine | 0.69 | 0.90 |
| | N-acetylglycine | 1.47 | 1.15 |
| | sarcosine | 0.84 | 1.48 |
| | dimethylglycine | 1.40 | 2.34 |
| | betaine | 0.85 | 1.55 |
| | serine | 0.87 | 1.02 |
| | N-acetylserine | 1.51 | 0.87 |
| | 2-methylserine | n.d. | 0.86 |
| | threonine | 1.16 | 1.17 |
| | N-acetylthreonine | 1.90 | 1.32 |
| | allo-threonine | n.d. | 1.07 |
| Alanine and Aspartate Metabolism | alanine | 1.41 | 2.01 |
| | N-acetylalanine | 1.48 | 1.41 |
| | N-carbamoylalanine | n.d. | 3.54 |
| | aspartate | 0.74 | 1.60 |
| | N-acetylaspartate (NAA) | 0.66 | 0.83 |
| | asparagine | 0.99 | 0.91 |
| | N-acetylasparagine | 1.15 | 1.19 |
| Glutamate Metabolism | glutamate | 0.94 | 2.20 |
| | glutamine | 0.68 | 1.97 |
| | N-acetylglutamate | 1.13 | 1.59 |
| | N-acetylglutamine | 1.11 | 1.87 |
| | 4-hydroxyglutamate | n.d. | 1.55 |
| | pyroglutamine\* | 0.94 | 0.98 |
| | N-acetyl-aspartyl-glutamate (NAAG) | n.d. | 0.76 |
| | beta-citrylglutamate | n.d. | 1.23 |
| | carboxyethyl-GABA | 1.34 | 1.38 |
| | S-1-pyrroline-5-carboxylate | 0.58 | 1.14 |
| Histidine Metabolism | histidine | 0.72 | 0.90 |
| | 1-methylhistidine | 0.70 | 1.27 |
| | 3-methylhistidine | 1.03 | 1.44 |
| | N-acetylhistidine | 1.07 | n.d. |
| | N-acetyl-1-methylhistidine\* | 0.33 | n.d. |
| | hydantoin-5-propionic acid | 4.69 | n.d. |
| | trans-urocanate | 1.32 | n.d. |
| | cis-urocanate | 0.96 | n.d. |
| | imidazole propionate | 2.28 | 1.95 |
| | formiminoglutamate | 3.29 | n.d. |
| | imidazole lactate | 1.41 | 0.58 |
| | carnosine | 2.00 | n.d. |
| | histamine | 0.18 | n.d. |
| | 1-methylhistamine | 0.48 | n.d. |
| | 1-methylimidazoleacetate | 1.22 | 1.00 |
| | 4-imidazoleacetate | 1.71 | 0.83 |
| Continued | | | |

## Slide 10
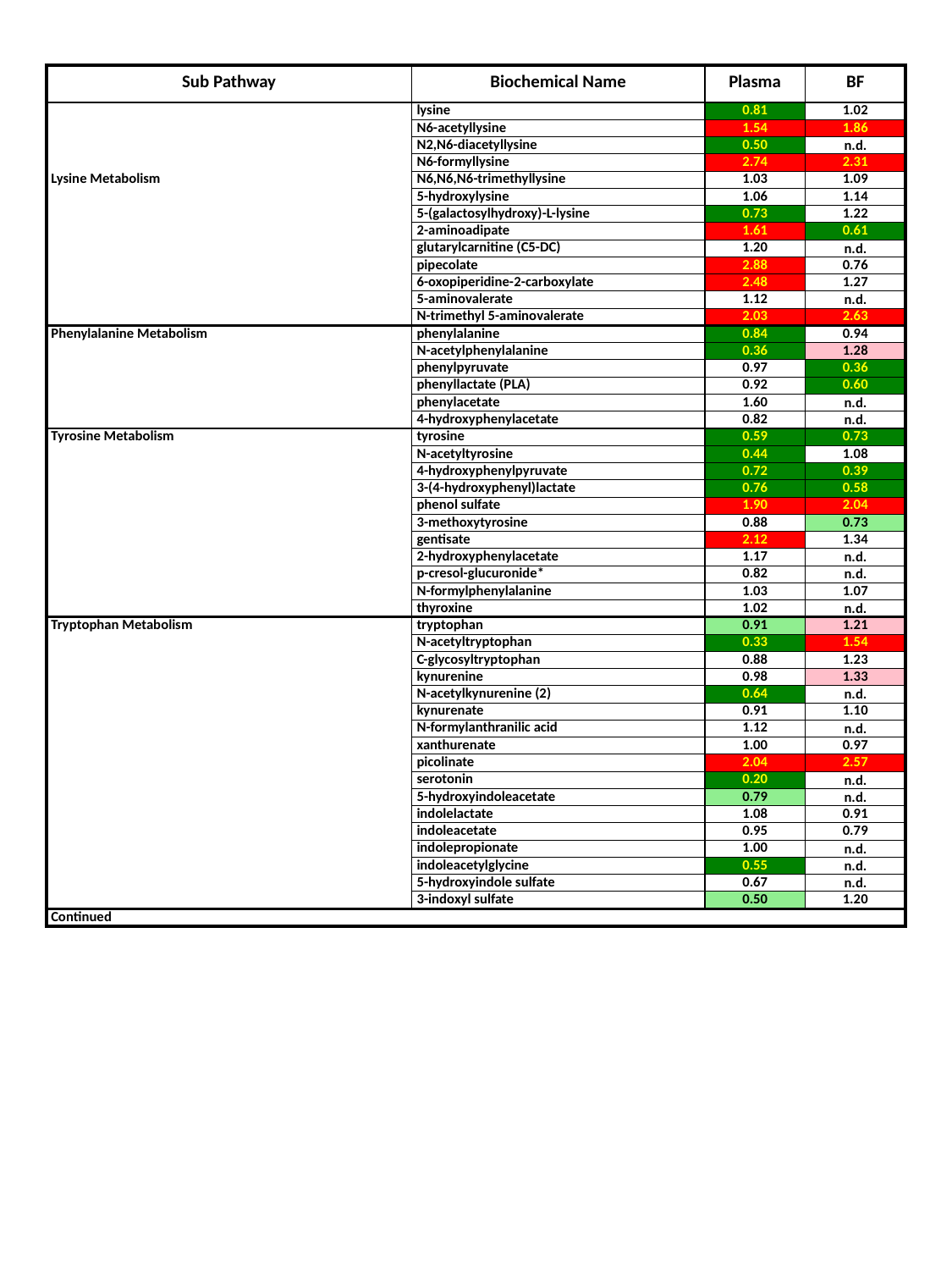

| Sub Pathway | Biochemical Name | Plasma | BF |
| --- | --- | --- | --- |
| | lysine | 0.81 | 1.02 |
| | N6-acetyllysine | 1.54 | 1.86 |
| | N2,N6-diacetyllysine | 0.50 | n.d. |
| | N6-formyllysine | 2.74 | 2.31 |
| Lysine Metabolism | N6,N6,N6-trimethyllysine | 1.03 | 1.09 |
| | 5-hydroxylysine | 1.06 | 1.14 |
| | 5-(galactosylhydroxy)-L-lysine | 0.73 | 1.22 |
| | 2-aminoadipate | 1.61 | 0.61 |
| | glutarylcarnitine (C5-DC) | 1.20 | n.d. |
| | pipecolate | 2.88 | 0.76 |
| | 6-oxopiperidine-2-carboxylate | 2.48 | 1.27 |
| | 5-aminovalerate | 1.12 | n.d. |
| | N-trimethyl 5-aminovalerate | 2.03 | 2.63 |
| Phenylalanine Metabolism | phenylalanine | 0.84 | 0.94 |
| | N-acetylphenylalanine | 0.36 | 1.28 |
| | phenylpyruvate | 0.97 | 0.36 |
| | phenyllactate (PLA) | 0.92 | 0.60 |
| | phenylacetate | 1.60 | n.d. |
| | 4-hydroxyphenylacetate | 0.82 | n.d. |
| Tyrosine Metabolism | tyrosine | 0.59 | 0.73 |
| | N-acetyltyrosine | 0.44 | 1.08 |
| | 4-hydroxyphenylpyruvate | 0.72 | 0.39 |
| | 3-(4-hydroxyphenyl)lactate | 0.76 | 0.58 |
| | phenol sulfate | 1.90 | 2.04 |
| | 3-methoxytyrosine | 0.88 | 0.73 |
| | gentisate | 2.12 | 1.34 |
| | 2-hydroxyphenylacetate | 1.17 | n.d. |
| | p-cresol-glucuronide\* | 0.82 | n.d. |
| | N-formylphenylalanine | 1.03 | 1.07 |
| | thyroxine | 1.02 | n.d. |
| Tryptophan Metabolism | tryptophan | 0.91 | 1.21 |
| | N-acetyltryptophan | 0.33 | 1.54 |
| | C-glycosyltryptophan | 0.88 | 1.23 |
| | kynurenine | 0.98 | 1.33 |
| | N-acetylkynurenine (2) | 0.64 | n.d. |
| | kynurenate | 0.91 | 1.10 |
| | N-formylanthranilic acid | 1.12 | n.d. |
| | xanthurenate | 1.00 | 0.97 |
| | picolinate | 2.04 | 2.57 |
| | serotonin | 0.20 | n.d. |
| | 5-hydroxyindoleacetate | 0.79 | n.d. |
| | indolelactate | 1.08 | 0.91 |
| | indoleacetate | 0.95 | 0.79 |
| | indolepropionate | 1.00 | n.d. |
| | indoleacetylglycine | 0.55 | n.d. |
| | 5-hydroxyindole sulfate | 0.67 | n.d. |
| | 3-indoxyl sulfate | 0.50 | 1.20 |
| Continued | | | |

## Slide 11
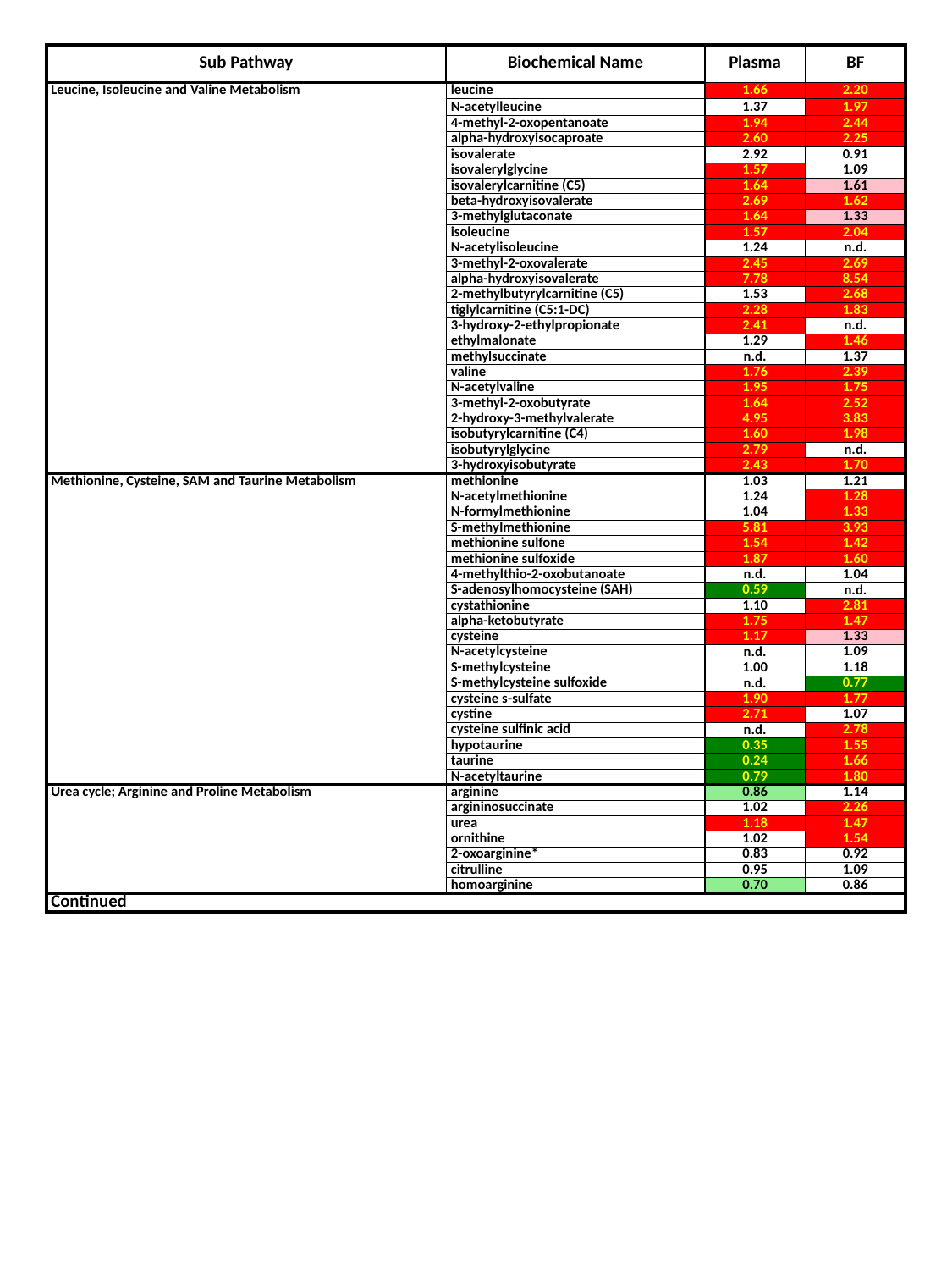

| Sub Pathway | Biochemical Name | Plasma | BF |
| --- | --- | --- | --- |
| Leucine, Isoleucine and Valine Metabolism | leucine | 1.66 | 2.20 |
| | N-acetylleucine | 1.37 | 1.97 |
| | 4-methyl-2-oxopentanoate | 1.94 | 2.44 |
| | alpha-hydroxyisocaproate | 2.60 | 2.25 |
| | isovalerate | 2.92 | 0.91 |
| | isovalerylglycine | 1.57 | 1.09 |
| | isovalerylcarnitine (C5) | 1.64 | 1.61 |
| | beta-hydroxyisovalerate | 2.69 | 1.62 |
| | 3-methylglutaconate | 1.64 | 1.33 |
| | isoleucine | 1.57 | 2.04 |
| | N-acetylisoleucine | 1.24 | n.d. |
| | 3-methyl-2-oxovalerate | 2.45 | 2.69 |
| | alpha-hydroxyisovalerate | 7.78 | 8.54 |
| | 2-methylbutyrylcarnitine (C5) | 1.53 | 2.68 |
| | tiglylcarnitine (C5:1-DC) | 2.28 | 1.83 |
| | 3-hydroxy-2-ethylpropionate | 2.41 | n.d. |
| | ethylmalonate | 1.29 | 1.46 |
| | methylsuccinate | n.d. | 1.37 |
| | valine | 1.76 | 2.39 |
| | N-acetylvaline | 1.95 | 1.75 |
| | 3-methyl-2-oxobutyrate | 1.64 | 2.52 |
| | 2-hydroxy-3-methylvalerate | 4.95 | 3.83 |
| | isobutyrylcarnitine (C4) | 1.60 | 1.98 |
| | isobutyrylglycine | 2.79 | n.d. |
| | 3-hydroxyisobutyrate | 2.43 | 1.70 |
| Methionine, Cysteine, SAM and Taurine Metabolism | methionine | 1.03 | 1.21 |
| | N-acetylmethionine | 1.24 | 1.28 |
| | N-formylmethionine | 1.04 | 1.33 |
| | S-methylmethionine | 5.81 | 3.93 |
| | methionine sulfone | 1.54 | 1.42 |
| | methionine sulfoxide | 1.87 | 1.60 |
| | 4-methylthio-2-oxobutanoate | n.d. | 1.04 |
| | S-adenosylhomocysteine (SAH) | 0.59 | n.d. |
| | cystathionine | 1.10 | 2.81 |
| | alpha-ketobutyrate | 1.75 | 1.47 |
| | cysteine | 1.17 | 1.33 |
| | N-acetylcysteine | n.d. | 1.09 |
| | S-methylcysteine | 1.00 | 1.18 |
| | S-methylcysteine sulfoxide | n.d. | 0.77 |
| | cysteine s-sulfate | 1.90 | 1.77 |
| | cystine | 2.71 | 1.07 |
| | cysteine sulfinic acid | n.d. | 2.78 |
| | hypotaurine | 0.35 | 1.55 |
| | taurine | 0.24 | 1.66 |
| | N-acetyltaurine | 0.79 | 1.80 |
| Urea cycle; Arginine and Proline Metabolism | arginine | 0.86 | 1.14 |
| | argininosuccinate | 1.02 | 2.26 |
| | urea | 1.18 | 1.47 |
| | ornithine | 1.02 | 1.54 |
| | 2-oxoarginine\* | 0.83 | 0.92 |
| | citrulline | 0.95 | 1.09 |
| | homoarginine | 0.70 | 0.86 |
| Continued | | | |

## Slide 12
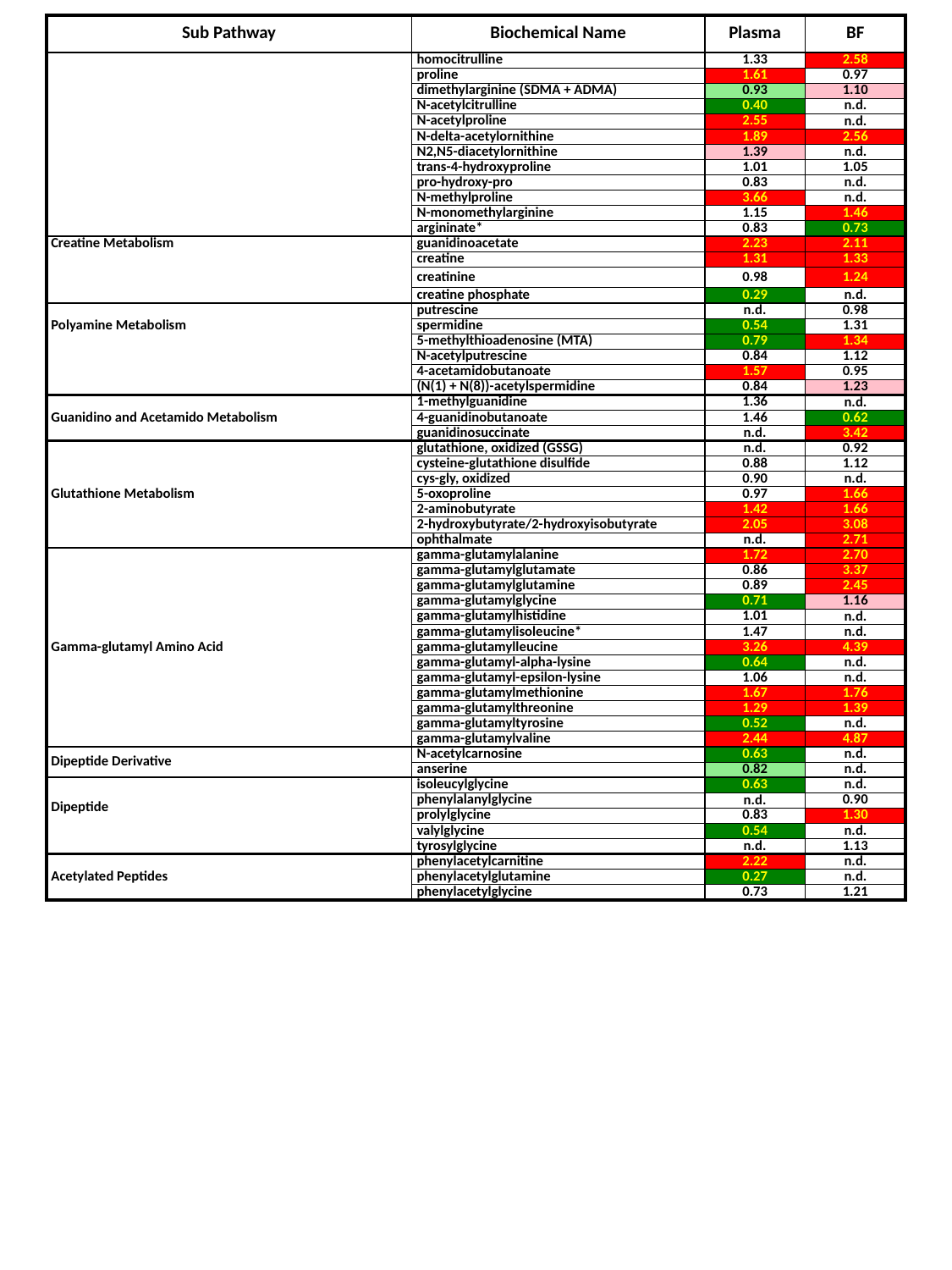

| Sub Pathway | Biochemical Name | Plasma | BF |
| --- | --- | --- | --- |
| | homocitrulline | 1.33 | 2.58 |
| | proline | 1.61 | 0.97 |
| | dimethylarginine (SDMA + ADMA) | 0.93 | 1.10 |
| | N-acetylcitrulline | 0.40 | n.d. |
| | N-acetylproline | 2.55 | n.d. |
| | N-delta-acetylornithine | 1.89 | 2.56 |
| | N2,N5-diacetylornithine | 1.39 | n.d. |
| | trans-4-hydroxyproline | 1.01 | 1.05 |
| | pro-hydroxy-pro | 0.83 | n.d. |
| | N-methylproline | 3.66 | n.d. |
| | N-monomethylarginine | 1.15 | 1.46 |
| | argininate\* | 0.83 | 0.73 |
| Creatine Metabolism | guanidinoacetate | 2.23 | 2.11 |
| | creatine | 1.31 | 1.33 |
| | creatinine | 0.98 | 1.24 |
| | creatine phosphate | 0.29 | n.d. |
| | putrescine | n.d. | 0.98 |
| Polyamine Metabolism | spermidine | 0.54 | 1.31 |
| | 5-methylthioadenosine (MTA) | 0.79 | 1.34 |
| | N-acetylputrescine | 0.84 | 1.12 |
| | 4-acetamidobutanoate | 1.57 | 0.95 |
| | (N(1) + N(8))-acetylspermidine | 0.84 | 1.23 |
| | 1-methylguanidine | 1.36 | n.d. |
| Guanidino and Acetamido Metabolism | 4-guanidinobutanoate | 1.46 | 0.62 |
| | guanidinosuccinate | n.d. | 3.42 |
| | glutathione, oxidized (GSSG) | n.d. | 0.92 |
| | cysteine-glutathione disulfide | 0.88 | 1.12 |
| | cys-gly, oxidized | 0.90 | n.d. |
| Glutathione Metabolism | 5-oxoproline | 0.97 | 1.66 |
| | 2-aminobutyrate | 1.42 | 1.66 |
| | 2-hydroxybutyrate/2-hydroxyisobutyrate | 2.05 | 3.08 |
| | ophthalmate | n.d. | 2.71 |
| Gamma-glutamyl Amino Acid | gamma-glutamylalanine | 1.72 | 2.70 |
| | gamma-glutamylglutamate | 0.86 | 3.37 |
| | gamma-glutamylglutamine | 0.89 | 2.45 |
| | gamma-glutamylglycine | 0.71 | 1.16 |
| | gamma-glutamylhistidine | 1.01 | n.d. |
| | gamma-glutamylisoleucine\* | 1.47 | n.d. |
| | gamma-glutamylleucine | 3.26 | 4.39 |
| | gamma-glutamyl-alpha-lysine | 0.64 | n.d. |
| | gamma-glutamyl-epsilon-lysine | 1.06 | n.d. |
| | gamma-glutamylmethionine | 1.67 | 1.76 |
| | gamma-glutamylthreonine | 1.29 | 1.39 |
| | gamma-glutamyltyrosine | 0.52 | n.d. |
| | gamma-glutamylvaline | 2.44 | 4.87 |
| Dipeptide Derivative | N-acetylcarnosine | 0.63 | n.d. |
| | anserine | 0.82 | n.d. |
| Dipeptide | isoleucylglycine | 0.63 | n.d. |
| | phenylalanylglycine | n.d. | 0.90 |
| | prolylglycine | 0.83 | 1.30 |
| | valylglycine | 0.54 | n.d. |
| | tyrosylglycine | n.d. | 1.13 |
| Acetylated Peptides | phenylacetylcarnitine | 2.22 | n.d. |
| | phenylacetylglutamine | 0.27 | n.d. |
| | phenylacetylglycine | 0.73 | 1.21 |

## Slide 13
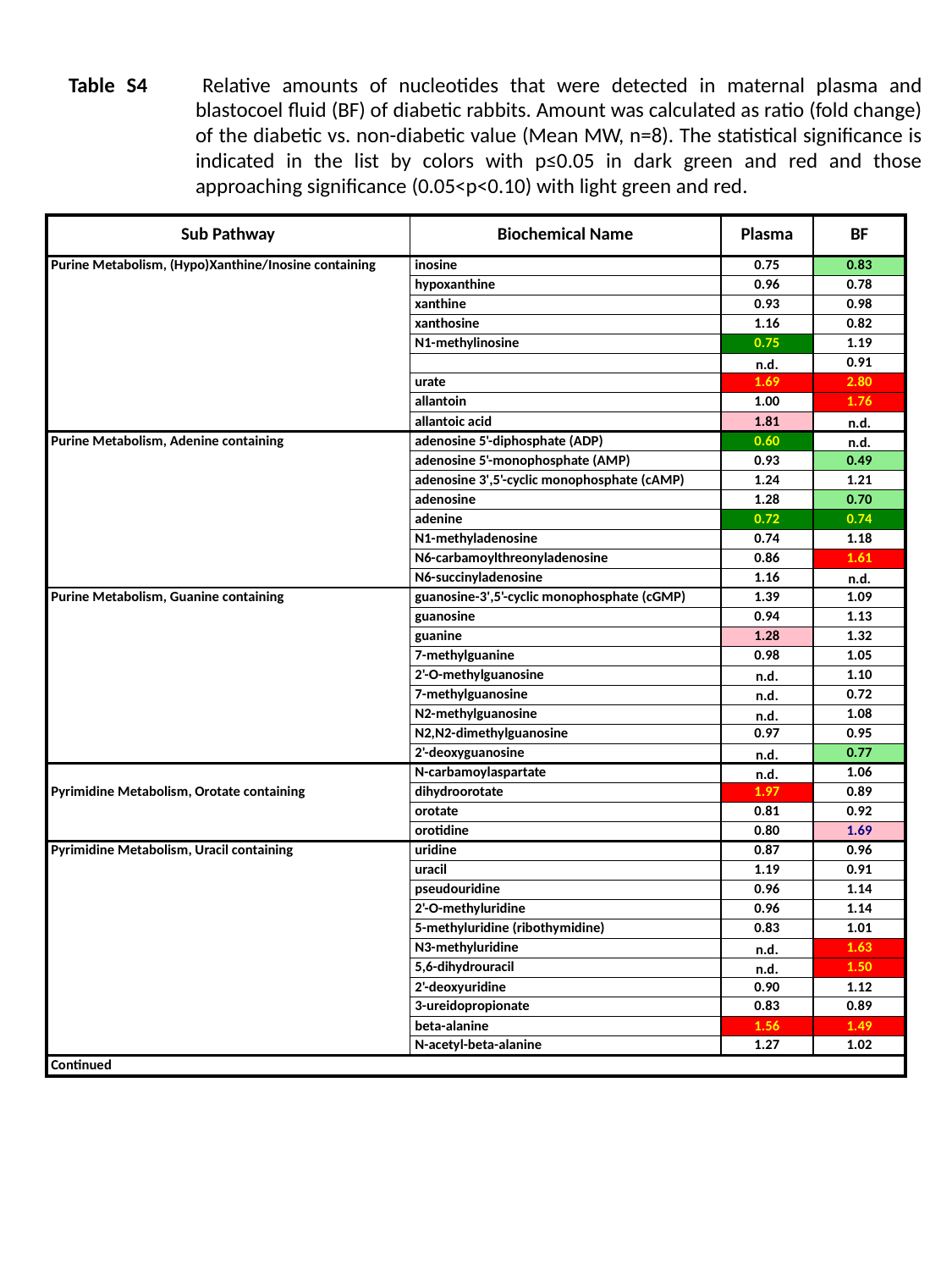

Table S4	Relative amounts of nucleotides that were detected in maternal plasma and 	blastocoel fluid (BF) of diabetic rabbits. Amount was calculated as ratio (fold change) 	of the diabetic vs. non-diabetic value (Mean MW, n=8). The statistical significance is 	indicated in the list by colors with p≤0.05 in dark green and red and those 	approaching significance (0.05<p<0.10) with light green and red.
| Sub Pathway | Biochemical Name | Plasma | BF |
| --- | --- | --- | --- |
| Purine Metabolism, (Hypo)Xanthine/Inosine containing | inosine | 0.75 | 0.83 |
| | hypoxanthine | 0.96 | 0.78 |
| | xanthine | 0.93 | 0.98 |
| | xanthosine | 1.16 | 0.82 |
| | N1-methylinosine | 0.75 | 1.19 |
| | | n.d. | 0.91 |
| | urate | 1.69 | 2.80 |
| | allantoin | 1.00 | 1.76 |
| | allantoic acid | 1.81 | n.d. |
| Purine Metabolism, Adenine containing | adenosine 5'-diphosphate (ADP) | 0.60 | n.d. |
| | adenosine 5'-monophosphate (AMP) | 0.93 | 0.49 |
| | adenosine 3',5'-cyclic monophosphate (cAMP) | 1.24 | 1.21 |
| | adenosine | 1.28 | 0.70 |
| | adenine | 0.72 | 0.74 |
| | N1-methyladenosine | 0.74 | 1.18 |
| | N6-carbamoylthreonyladenosine | 0.86 | 1.61 |
| | N6-succinyladenosine | 1.16 | n.d. |
| Purine Metabolism, Guanine containing | guanosine-3',5'-cyclic monophosphate (cGMP) | 1.39 | 1.09 |
| | guanosine | 0.94 | 1.13 |
| | guanine | 1.28 | 1.32 |
| | 7-methylguanine | 0.98 | 1.05 |
| | 2'-O-methylguanosine | n.d. | 1.10 |
| | 7-methylguanosine | n.d. | 0.72 |
| | N2-methylguanosine | n.d. | 1.08 |
| | N2,N2-dimethylguanosine | 0.97 | 0.95 |
| | 2'-deoxyguanosine | n.d. | 0.77 |
| | N-carbamoylaspartate | n.d. | 1.06 |
| Pyrimidine Metabolism, Orotate containing | dihydroorotate | 1.97 | 0.89 |
| | orotate | 0.81 | 0.92 |
| | orotidine | 0.80 | 1.69 |
| Pyrimidine Metabolism, Uracil containing | uridine | 0.87 | 0.96 |
| | uracil | 1.19 | 0.91 |
| | pseudouridine | 0.96 | 1.14 |
| | 2'-O-methyluridine | 0.96 | 1.14 |
| | 5-methyluridine (ribothymidine) | 0.83 | 1.01 |
| | N3-methyluridine | n.d. | 1.63 |
| | 5,6-dihydrouracil | n.d. | 1.50 |
| | 2'-deoxyuridine | 0.90 | 1.12 |
| | 3-ureidopropionate | 0.83 | 0.89 |
| | beta-alanine | 1.56 | 1.49 |
| | N-acetyl-beta-alanine | 1.27 | 1.02 |
| Continued | | | |

## Slide 14
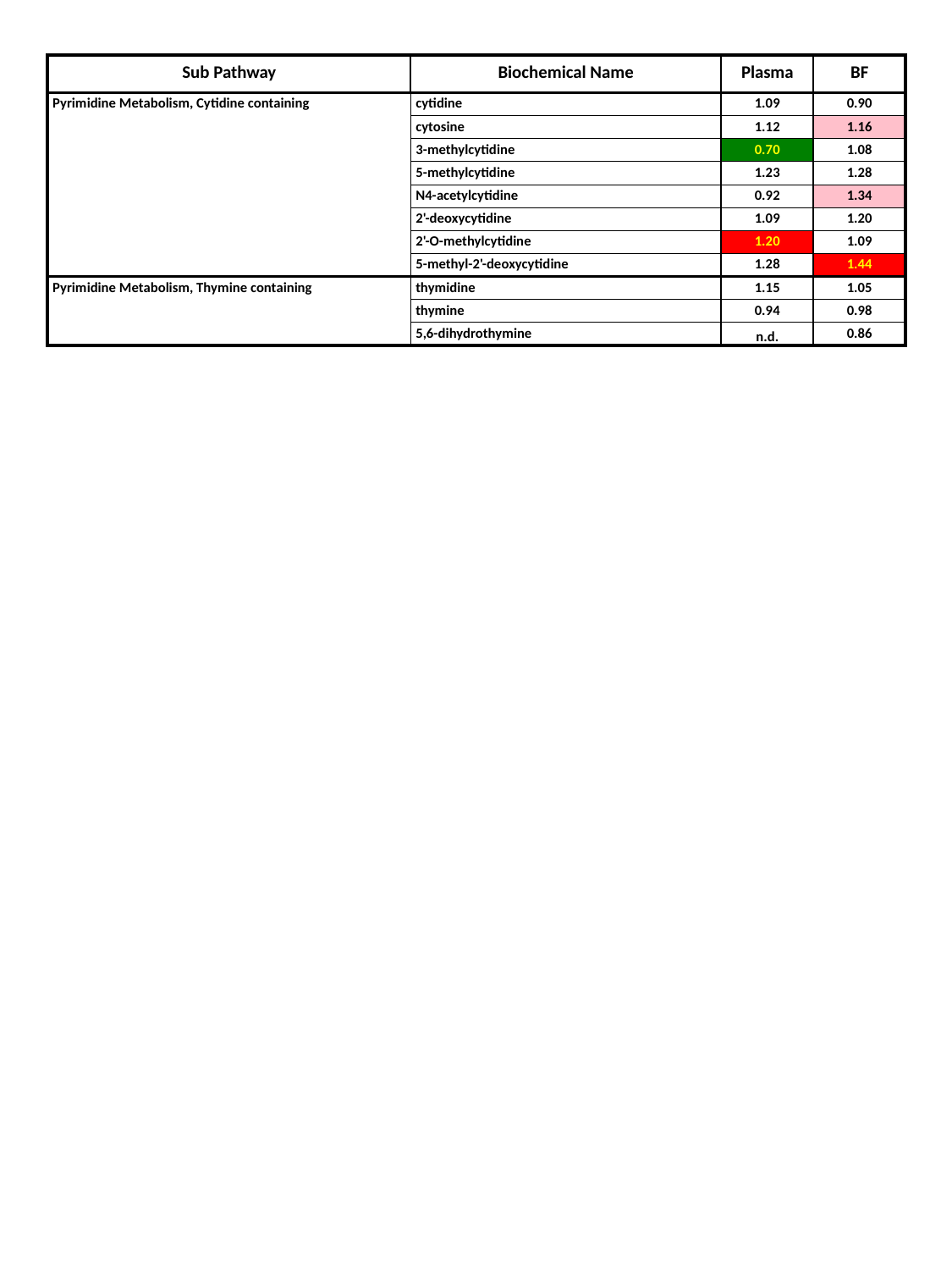

| Sub Pathway | Biochemical Name | Plasma | BF |
| --- | --- | --- | --- |
| Pyrimidine Metabolism, Cytidine containing | cytidine | 1.09 | 0.90 |
| | cytosine | 1.12 | 1.16 |
| | 3-methylcytidine | 0.70 | 1.08 |
| | 5-methylcytidine | 1.23 | 1.28 |
| | N4-acetylcytidine | 0.92 | 1.34 |
| | 2'-deoxycytidine | 1.09 | 1.20 |
| | 2'-O-methylcytidine | 1.20 | 1.09 |
| | 5-methyl-2'-deoxycytidine | 1.28 | 1.44 |
| Pyrimidine Metabolism, Thymine containing | thymidine | 1.15 | 1.05 |
| | thymine | 0.94 | 0.98 |
| | 5,6-dihydrothymine | n.d. | 0.86 |

## Slide 15
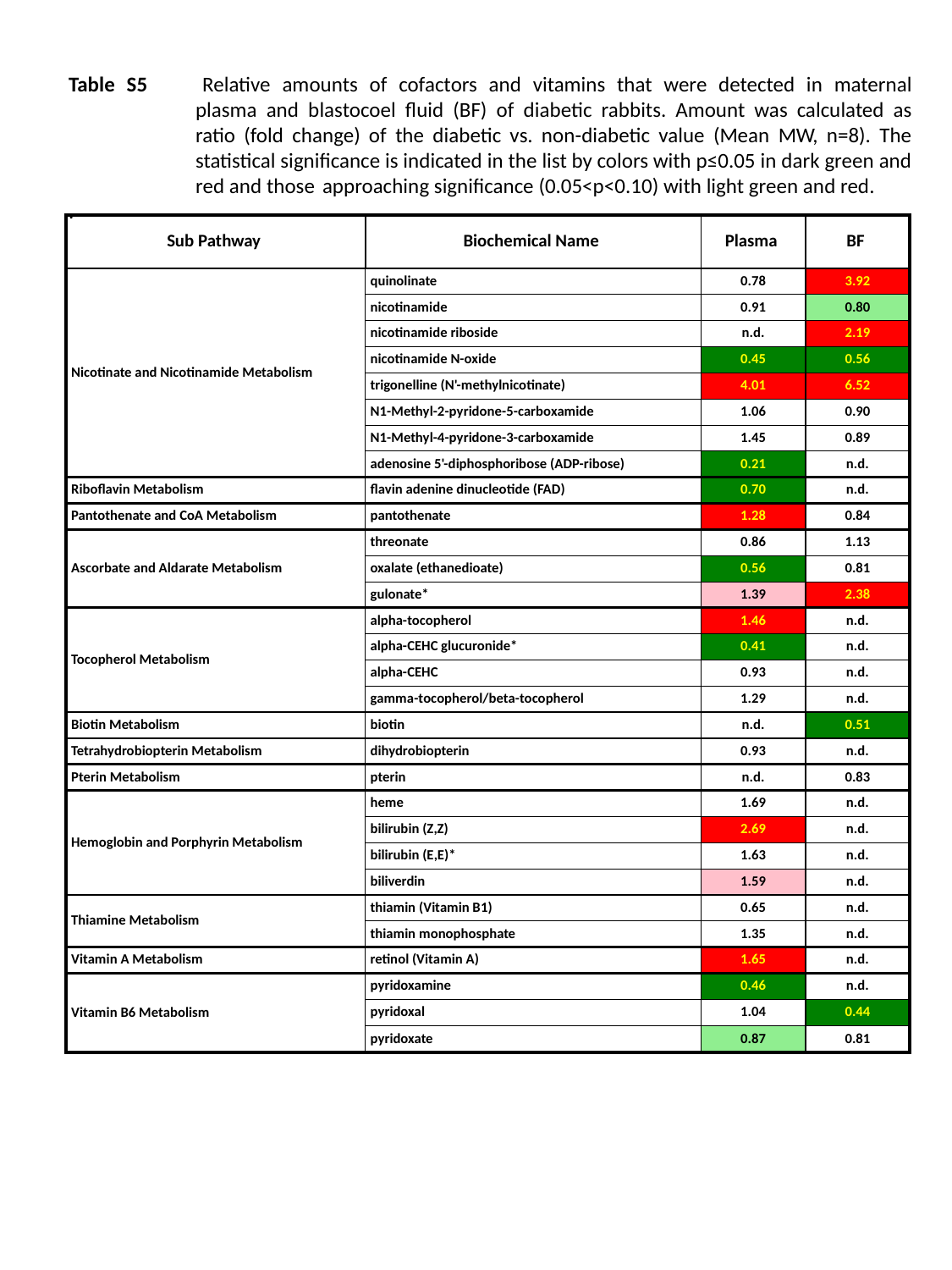

Table S5	Relative amounts of cofactors and vitamins that were detected in maternal 	plasma and blastocoel fluid (BF) of diabetic rabbits. Amount was calculated as 	ratio (fold change) of the diabetic vs. non-diabetic value (Mean MW, n=8). The 	statistical significance is indicated in the list by colors with p≤0.05 in dark green and 	red and those 	approaching significance (0.05<p<0.10) with light green and red.
.
| Sub Pathway | Biochemical Name | Plasma | BF |
| --- | --- | --- | --- |
| Nicotinate and Nicotinamide Metabolism | quinolinate | 0.78 | 3.92 |
| | nicotinamide | 0.91 | 0.80 |
| | nicotinamide riboside | n.d. | 2.19 |
| | nicotinamide N-oxide | 0.45 | 0.56 |
| | trigonelline (N'-methylnicotinate) | 4.01 | 6.52 |
| | N1-Methyl-2-pyridone-5-carboxamide | 1.06 | 0.90 |
| | N1-Methyl-4-pyridone-3-carboxamide | 1.45 | 0.89 |
| | adenosine 5'-diphosphoribose (ADP-ribose) | 0.21 | n.d. |
| Riboflavin Metabolism | flavin adenine dinucleotide (FAD) | 0.70 | n.d. |
| Pantothenate and CoA Metabolism | pantothenate | 1.28 | 0.84 |
| Ascorbate and Aldarate Metabolism | threonate | 0.86 | 1.13 |
| | oxalate (ethanedioate) | 0.56 | 0.81 |
| | gulonate\* | 1.39 | 2.38 |
| Tocopherol Metabolism | alpha-tocopherol | 1.46 | n.d. |
| | alpha-CEHC glucuronide\* | 0.41 | n.d. |
| | alpha-CEHC | 0.93 | n.d. |
| | gamma-tocopherol/beta-tocopherol | 1.29 | n.d. |
| Biotin Metabolism | biotin | n.d. | 0.51 |
| Tetrahydrobiopterin Metabolism | dihydrobiopterin | 0.93 | n.d. |
| Pterin Metabolism | pterin | n.d. | 0.83 |
| Hemoglobin and Porphyrin Metabolism | heme | 1.69 | n.d. |
| | bilirubin (Z,Z) | 2.69 | n.d. |
| | bilirubin (E,E)\* | 1.63 | n.d. |
| | biliverdin | 1.59 | n.d. |
| Thiamine Metabolism | thiamin (Vitamin B1) | 0.65 | n.d. |
| | thiamin monophosphate | 1.35 | n.d. |
| Vitamin A Metabolism | retinol (Vitamin A) | 1.65 | n.d. |
| Vitamin B6 Metabolism | pyridoxamine | 0.46 | n.d. |
| | pyridoxal | 1.04 | 0.44 |
| | pyridoxate | 0.87 | 0.81 |

## Slide 16
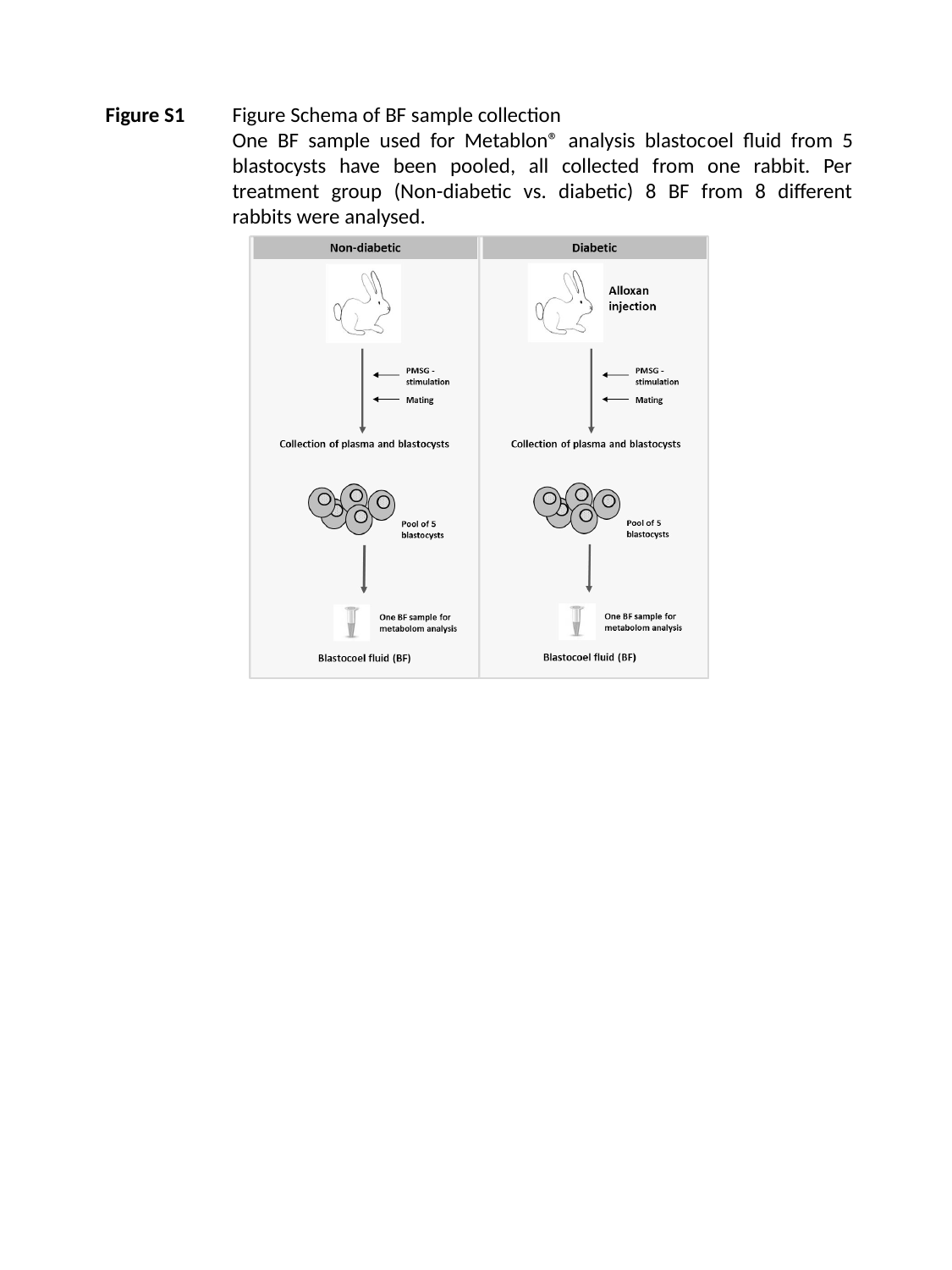

Figure S1	Figure Schema of BF sample collection
	One BF sample used for Metablon® analysis blastocoel fluid from 5 	blastocysts have been pooled, all collected from one rabbit. Per 	treatment group (Non-diabetic vs. diabetic) 8 BF from 8 different 	rabbits were analysed.
